# Factors associated with intended use of a maternity waiting home in Southern Ethiopia: a community-based cross-sectional study

**DOI:** 10.1186/s12884-018-1670-z

**Published:** 2018-01-19

**Authors:** Tienke Vermeiden, Floris Braat, Girmay Medhin, Asheber Gaym, Thomas van den Akker, Jelle Stekelenburg

**Affiliations:** 1 Butajira General Hospital, Butajira, Southern Nations, Nationalities and Peoples’ Region Ethiopia; 2 Department of Health Sciences, Global Health, University Medical Centre / University of Groningen, Groningen, The Netherlands; 3 Aklilu Lemma Institute of Pathobiology, Addis Ababa University, Addis Ababa, Ethiopia; 4 Unicef Ethiopia, Addis Ababa, Ethiopia; 5 Department of Obstetrics, Leiden University Medical Centre, Leiden, The Netherlands; 6 Department of Obstetrics and Gynaecology, Leeuwarden Medical Centre, Leeuwarden, The Netherlands; 7 Bikita District, Zimbabwe

**Keywords:** Maternity waiting homes, Maternal health, Maternal mortality, Obstetrics, Health-seeking behaviour, Ethiopia

## Abstract

**Background:**

Although Ethiopia is scaling up Maternity Waiting Homes (MWHs) to reduce maternal and perinatal mortality, women’s use of MWHs varies markedly between facilities. To maximize MWH utilization, it is essential that policymakers are aware of supportive and inhibitory factors. This study had the objective to describe factors and perceived barriers associated with potential utilization of an MWH among recently delivered and pregnant women in Southern Ethiopia.

**Methods:**

A community-based cross-sectional study was conducted between March and November 2014 among 428 recently delivered and pregnant women in the Eastern Gurage Zone, Southern Ethiopia, where an MWH was established for high-risk pregnant women to await onset of labour. The structured questionnaire contained questions regarding possible determinants and barriers. Logistic regression with 95% Confidence Intervals (CI) was used to examine association of selected variables with potential MWH use.

**Results:**

While only thirty women (7.0%) had heard of MWHs prior to the study, 236 (55.1%), after being explained the concept, indicated that they intended to stay at such a structure in the future. The most important factors associated with intended MWH use in the bivariate analysis were a woman’s education (secondary school or higher vs. no schooling: odds ratio [OR] 6.3 [95% CI 3.46 to 11.37]), her husband’s education (secondary school or higher vs. no schooling: OR 5.4 [95% CI 3.21 to 9.06]) and envisioning relatively few barriers to MWH use (OR 0.32 [95% CI 0.25 to 0.39]). After adjusting for possible confounders, potential users had more frequently suffered complications in previous childbirths (adjusted odds ratio [aOR] 4.0 [95% CI 1.13 to 13.99]) and envisioned fewer barriers to MWH use (aOR 0.3 [95% CI 0.23 to 0.38]). Barriers to utilization included being away from the household (aOR 18.1 [95% CI 5.62 to 58.46]) and having children in the household cared for by the community during a woman’s absence (aOR 9.3 [95% CI 2.67 to 32.65]).

**Conclusions:**

Most respondents had no knowledge about MWHs. Having had complications during past births and envisioning few barriers were factors found to be positively associated with intended MWH use. Unless community awareness of preventive maternity care increases and barriers for women to stay at MWHs are overcome, these facilities will continue to be underutilized, especially among marginalized women.

**Electronic supplementary material:**

The online version of this article (10.1186/s12884-018-1670-z) contains supplementary material, which is available to authorized users.

## Background

Globally, the Maternal Mortality Ratio fell by 44% between 1990 and 2015, from approximately 385 to 216 per 100,000 live births. Despite this reduction, more than 300,000 pregnant and recently delivered women still died in 2015, of whom 200,000 in sub-Saharan Africa [1]. More than 80% of these deaths could have been prevented by appropriate, timely interventions, performed by skilled professionals in a conducive environment [2–4].

Ethiopia’s Maternal Mortality Ratio dropped from 676 to 412 per 100,000 live births between 2011 and 2016, although – in absolute numbers – Ethiopia was still among the top-five countries contributing to global maternal mortality in 2013 [1, 5]. In 2016, only 26% of women in Ethiopia reported that they had given birth to their last-born at a health facility. This rate is among the lowest in the world [5, 6]. Reasons include notions that facility delivery is not necessary or customary, as well as physical distance to the facility and lack of transportation [7].

Maternity Waiting Homes (MWHs) are built to reduce delays in reaching health facilities in time. An MWH is a structure within easy reach of an emergency obstetric and new-born care facility, where women with high-risk pregnancies await onset of labour during the final weeks of pregnancy. This includes women from remote areas, grand multiparous women (those who have given birth five times or more), and women with scarred uteri, multiple pregnancies or a previous stillbirth [3, 8, 9].

At this point in time, evidence for a positive effect of MWHs on maternal and neonatal outcomes is limited, although several studies have reported positive results [2]. The largest observational study was conducted in Ethiopia, and found substantially lower maternal mortality and stillbirth rates among women who had been admitted into an MWH compared to those who were admitted directly to hospital [10].

Since 2014, the Ethiopian Ministry of Health has implemented MWHs throughout the country [11]. Around the time of this study, Ethiopia had nine such MWHs. Some of these received many women, while others remained empty [9]. Several, mostly qualitative, studies from other settings have examined such limited use of MWH services, and have underlined the need to take local customs and practice, and other supportive and inhibitory factors into account when planning to establish an MWH [2]. This study was undertaken to describe facilitating factors and perceived barriers associated with potential utilization of an MWH. Results from this study may advise policy-makers as to which factors they should consider when implementing the MWH program in Ethiopia.

## Methods

A community-based cross-sectional study was undertaken between March and November 2014 among women who had given birth in the three years prior to the study or who were pregnant at the time of the study. Participants had to be able to communicate in the national language Amharic. Ethical approval for the study was obtained from the Southern Nations Nationalities and People Regional State Health Bureau in Hawassa, Ethiopia.

The study took place in the Eastern Gurage Zone, a predominantly rural area in the Southern Nations Nationalities and People Regional State of Ethiopia, with an estimated population of over 500,000. Administratively, this zone is divided into four districts: Meskan, Mareko, Soddo, and Butajira town (the largest urbanized area), with approximately 46,000, 19,000, 42,000 and 11,000 women in the reproductive age group, respectively (personal communication from Zonal Health Bureau Welkite, 2 May 2015). While no regional data are available to calculate a Crude Birth Rate (CBR) for the area, the national CBR was 32 births per 1000 people in 2016 [5]. A total of 119 health posts, 20 health centres and two general hospitals (one governmental and one faith-based) served the population. The closest tertiary referral hospital was in the capital, Addis Ababa, at approximately two hours’ distance by ambulance. At the time of the study, ambulances were available at hospital and district level, but these had difficulties accessing remote areas, particularly in the rainy season. Project Mercy Hospital, located in a small village near Butajira town, opened an MWH in 2012.

This study was conducted for Butajira General Hospital, located in Butajira town, which established an MWH on its grounds in 2015. At the time of the study, the hospital had a catchment area population of around one million, serving people from the Eastern Gurage Zone and neighbouring zones. Butajira Hospital provides 24-h comprehensive emergency obstetric and new-born care and the number of births was approximately 3000 in 2014. Delivery services became free-of-charge in the first quarter of 2014. Access to the MWH is provided at no cost.

A sample size of 383 was calculated using Epi Info StatCalc, with a 5% margin of error and a 95% confidence interval (CI), by using the estimated number of women in the reproductive age group in the Eastern Gurage Zone (118,000). Since the true rate of expected MWH-use was unknown, the expected frequency was set at 50%, which gives the largest possible sample size. A design effect of 1.0 was used. In total, 428 respondents were conveniently sampled from each of the four districts in the Eastern Gurage Zone: 120 women from Butajira town, 108 from Meskan district, 100 from Soddo district, and 100 from Mareko district. In Butajira, participants were selected from each of the five ‘Kebeles’ or neighbourhoods. In the other three districts, data collection took place near health centres. Five health centres were randomly selected from Meskan and Soddo and four from the smallest district Mareko. In Soddo, one of the randomly selected health centres was not accessible by public transport at that time, which led us to purposely choosing another centre at a similar distance. Participants were selected by visiting every third household. If a woman in that household had given birth in the three years prior to or was pregnant at the time of the survey, and could communicate in the national language Amharic, she was asked to participate. Whenever more than one eligible woman was found in the same household, one was randomly selected and included in the study. If no one in that specific household fulfilled the inclusion criteria, the neighbouring house was visited. One woman declined participation, stating that she needed permission from her husband, who was not available. Informed written consent was obtained from all participants, through their signature or fingerprint.

Variables were formulated using the Adapted Three Delay Model, which describes possible delays in (1) deciding to seek birth care, (2) trying to identify and reach a health facility, and (3) receiving adequate and appropriate treatment. This Adapted Three Delay Model was formulated by Gabrysch et al., who expanded the original model arguing that the latter implicitly considers home births with complications and that (possibly reduced) delays of what the authors call “preventive facility births” should be more explicitly included in the model. These factors are grouped into four themes: sociocultural factors, perceived need/benefit, economic and physical accessibility [12, 13]. A structured questionnaire was developed in English, translated into the national language Amharic and then translated back into English to check for consistency [see Additional files 1 and 2 for the English and Amharic versions of the questionnaire]. All questions except those concerning *relative household wealth*, *likelihood of staying at an MWH* and *envisioned barriers to MWH use* were taken from the Ethiopian Demographic Health Survey [14]. Questions regarding the use of an MWH were formulated based on a previous Ethiopian study [9]. The questionnaire was pre-tested twice among pregnant and recently delivered women in Butajira Hospital, first by a medical doctor and thereafter by a data collection team. They read questionnaires out loud and completed these in the presence of an observer, which led to improvements in the questionnaire’s layout and explanatory texts.

Data collection with regard to socio-cultural factors comprised of the respondent’s estimated *age category*, *marital status*, and *her and her husband’s educational level*. *Decision-making power* was determined by a combined score of answers to two questions regarding who is involved in deciding on family earnings and in matters of maternal and child health. Women who generally made decisions independently or jointly with their husbands were considered to have decision-making power. *Health education* is one of the included factors relating to perceived need/benefit: women were asked if they had received information about signs of pregnancy complications during antenatal care visits and if they could name any (eight options were provided for the data collector: vaginal bleeding, vaginal flush of fluid, severe headache, blurred vision, fever, abdominal pain/preterm contractions, decreased foetal movement, oedema/body swelling, plus the option ‘other, specify…’). *Parity* was defined as the number of times a woman had given birth, including intrauterine deaths and stillbirths. *History of facility delivery* was recorded as ‘birthing location’*.* Primigravida were recorded as not having a history of home or facility birth. If a respondent’s births all took place at the same location, the last birth was explored in terms of the reason(s) why she delivered at home or at a facility and, if applicable, which *complications* she suffered. If there had been a change in birthing location, we prompted for reasons why she had previously given birth both at home and at the facility, and, if applicable, which complications she had suffered. Answers were recorded using a multiple response set: haemorrhage, prolonged labour, obstructed labour, hypertensive disorder, puerperal infection, foetal distress, intrauterine foetal death, and ‘other, namely’. For the analyses, *complications* were clustered into a yes-no score. Economic accessibility was assessed by asking respondents to compare the *wealth* of their household with those around them on a four-point scale (very wealthy, wealthy, poor, very poor). In the analyses, a combined score was used. Physical accessibility was defined by a respondent’s *travel time* from her household to the nearest hospital. *Urban/rural residency* was based on the 2007 Population Census [15]. The questionnaire contained specific questions regarding the *likelihood of staying at an MWH* and *perceived social and economic barriers* to using an MWH. First, respondents were asked if they had ever heard of an MWH. Regardless of their answers, they were then explained the concept of an MWH: “A Maternity Waiting Home is a place for high-risk pregnant women to await birth in their last weeks of pregnancy, close to 24/7 emergency obstetric care. Possible reasons to stay are for example a previous caesarean section or haemorrhage, previous stillbirth or neonatal death, breech presentation, twin pregnancy, or living far from a hospital.” Respondents were then asked if they knew an MWH in the region and if they believed there were advantages to staying at an MWH and if so, what these advantages would be. Subsequently they were asked how likely it would be for them to stay at an MWH during the last two to four weeks of their current or next pregnancy using a four-point scale (very likely, likely, unlikely, very unlikely). In the analyses, a combined score was used. Finally, respondents were asked to imagine staying at an MWH for two to four weeks and how they might arrange transport and food, bring their own cooking utensils, stay for that length of time, bring an attendant to accompany them, and arrange for others at home to take care of their children and household chores. Envisioned barriers were measured with a dichotomous scale (possible/affordable, not possible/not affordable).

The data collection team comprised of one female supervisor and five female data collectors from Butajira town who completed at least ten years of education. Data were entered by two staff members. All field research staff enrolled in a two-day training that included study objectives, topics related to maternal health, interviewing skills, role-play, and test questionnaires. For the data-entry staff, specific training was given on SPSS.

Completed questionnaires were checked for completeness in the field and households were revisited to complement incomplete data. Quantitative data were then computerized using SPSS 22. Subsequently, all data were double-checked variable by variable and cross-checked between variables by the primary investigator (TV). To investigate which factors were associated with the intention to use the MWH intervention, the sample was divided into two nominal categories: (1) women who indicated they were unlikely to use an MWH (“Potential Non-Users”) and (2) women who stated they were likely to use one (“Potential Users”). In our analyses, these categories are the outcome of interest. Variables were selected based on a literature review, considering their importance in the Ethiopian setting (e.g. previous facility delivery, previous complications), previously found associations in various directions (e.g. decision-making power), and/or to be able to adjust for potential confounders (e.g. maternal age, wealth). Bivariate and multivariable logistic regression analyses were performed using all selected variables from the Adapted Three Delay Model, in order to investigate which of these (women’s decision-making power, previous place of delivery, etc.) influence the outcome of interest. Women with missing responses in any of the selected variables were excluded from multivariable regression. The proportions were calculated using the total number of respondents. Due to some missing responses, percentages will not always add up to 100.0%.

Envisioned social and economic barriers of using an MWH (transport to and from an MWH, arranging your own food at an MWH, having to bring your own cooking utensils, etc.) were included separately in a model, to show which of these possible barriers have the greatest influence on the likelihood of utilizing an MWH during the current or next pregnancy. Proportions were calculated using the total number of Potential Users and Potential Non-Users.

Using logistic regression, crude Odds Ratio (OR) and adjusted Odds Ratio (aOR) with 95% CI were calculated to measure the effect of each independent variable on the target outcome variable.

## Results

Of all 428 respondents, 419 (97.9%) were married. On average, women had given birth 2.8 (±1.91) times. Thirty women (7.0%) had heard of an MWH prior to the survey. After learning about the MWH concept, 236 (55.1%) of all women indicated they would be likely to stay at one during their current or next pregnancy. Table 1 lists those factors that make MWH use more likely. In the bivariate analysis, each of the selected variables was associated with Potential MWH use, except for being in the age range of 25 to 29, parity and having had complications in previous births. Potential Users were more often below 25 years of age, educated, and had higher decision-making power. They were also more likely to have husbands with higher educational levels. Likewise, women who had received health education about danger signs of pregnancy complications, had had one or more previous facility-based childbirths, and envisioned few barriers to staying at an MWH had higher odds of being a Potential User. On average, these women were relatively wealthy, lived closer to hospitals, and in urban areas. Data of 407 respondents (95%) were included for multiple regression. Table 1 shows that after adjusting for possible confounding variables, Potential Users more frequently had suffered complications in previous childbirths and envisioned fewer barriers to using an MWH.

**Table 1:** Factors associated with potential utilization of a Maternity Waiting Home (*N* = 428)

Variables & categories	Overall (*N* = 428)	Potential MWH Users (*n* = 236)	Potential Non-MWH Users (*n* = 190)	Odds Ratio 95% CI	Adjusted Odds Ratio 95% CI
Age (in years)					
≥ 30	157 (36.7)	77 (32.9)	80 (42.1)	1	1
25–29	182 (42.5)	100 (42.4)	81 (42.6)	1.28 (0.84–1.97)	0.74 (0.25–2.20)
≤ 24	89 (20.8)	59 (25.0	29 (15.3)	2.11 (1.23–3.64)*	1.92 (0.43–8.62)
Educational level					
No schooling	181 (42.3)	72 (30.5)	107 (56.3)	1	1
Primary school	153 (35.7)	88 (37.3)	65 (34.2)	2.01 (1.30–3.12)*	0.64 (0.21–1.92)
Secondary school and higher	94 (22.0)	76 (32.2)	18 (9.5)	6.28 (3.46–11.37)*	0.36 (0.07–1.95)
Husband’s educational level					
No schooling	125 (29.2)	43 (18.2)	80 (42.3)	1	1
Primary school	150 (35.1)	80 (33.9)	70 (36.8)	2.13 (1.30–3.47)*	1.44 (0.46–4.52)
Secondary school and higher	152 (35.5)	113 (47.9)	39 (20.5)	5.39 (3.21–9.06)*	2.40 (0.60–9.54)
Decision-making power					
No	183 (42.8)	82 (35.7)	100 (52.9)	1	1
Yes	240 (56.1)	148 (64.3)	89 (47.0)	2.06 (1.39–3.04)*	1.66 (0.65–4.27)
Health education danger signs					
No	215 (50.2)	94 (39.8)	120 (63.2)	1	1
Yes	213 (49.8)	142 (60.2)	70 (36.8)	2.59 (1.75–3.84)*	1.41 (0.56–3.60)
Parity					
0 births	28 (6.5)	9 (4.7)	19 (8.1)	1	1
1–4 births	316 (73.8)	137 (72.1)	177 (75.0)	0.61 (0.27–1.40)	0.76 (0.10–5.85)
≥ 5 births	84 (19.6)	44 (23.2)	40 (16.9)	0.43 (0.18–1.06)	1.24 (0.11–13.87)
History of health facility delivery					
No	163 (38.1)	68 (28.9)	94 (49.7)	1	1
Yes	141 (32.9)	84 (35.7)	57 (30.2)	2.04 (1.29–3.22)*	0.84 (0.21–3.33)
Two or more	122 (28.5)	83 (35.3)	38 (20.1)	3.02 (1.84–4.95)*	1.82 (0.47–7.06)
History of complications					
No	344 (80.4)	187 (80.6)	155 (82.4)	1	1
Yes	78 (18.2)	45 (19.4)	33 (17.6)	1.13 (0.69–1.86)	3.98 (1.13–13.99)*
Relative household wealth					
(Very) poor	270 (63.1)	122 (51.7)	146 (76.8)	1	1
(Very) wealthy	158 (36.9)	114 (48.3)	44 (23.2)	3.10 (2.03–4.73)*	0.77 (0.27–2.23)
Travel time to nearest hospital (in minutes)					
> 60	147 (34.3)	67 (28.4)	79 (41.6)	1	1
30–60	207 (48.4)	120 (50.8)	86 (45.3)	1.65 (1.07–2.52)*	0.97 (0.24–3.89)
< 30	74 (17.3)	49 (20.8)	25 (13.2)	2.31 (1.29–4.13)*	1.49 (0.24–9.14)
Region					
Rural	217 (50.7)	97 (41.1)	118 (62.1)	1	1
Urban	211 (49.3)	139 (58.9)	72 (37.9)	2.35 (1.59–3.47)*	0.99 (0.23–4.28)
Envisioned barriers MWH utilization (mean ± SD; *n* = 422, range 0–9)	3.59 ± 3.319	1.08 ± 1.604	6.67 ± 2.021	0.32 (0.25–0.39)*	0.30 (0.23–0.38)*

*p < 0.05

Table 2 shows perceived barriers to potential utilization of an MWH. All these barriers were associated with lack of willingness to use an MWH in the future. Data for 409 (96%) respondents were included for multiple regression. The adjusted model indicates that Potential Non-Users more often considered it impossible for them to be away from their household for two to four weeks prior to the due date. Another significant barrier to use was having to rely on family or community members to take care of other children during a woman’s absence. Furthermore, the odds of being a Potential Non-User were higher among women who considered it impossible for a family or community member to accompany them to the MWH as “attendants” and women who could not afford transport to and from an MWH.

**Table 2:** Envisioned barriers associated with utilization of a Maternity Waiting Home (*N* = 428)

Variables & categories	Overall (*N* = 428)	Potential MWH Users (*n* = 236)	Potential Non-MWH Users (*n* = 190)	Odds Ratio 95% CI	Adjusted Odds Ratio 95% CI
Transport to and from the MWH					
Not affordable	143 (33.4)	24 (10.2)	118 (62.1)	1	1
Affordable	283 (66.1)	211 (89.8)	72 (37.9)	14.41 (8.62–24.09)*	3.61 (1.04–12.46)*
Food while staying at MWH					
Not affordable	179 (41.8)	38 (16.2)	140 (74.1)	1	1
Affordable	246 (57.5)	197 (83.8)	49 (25.9)	14.81 (9.20–23.84)*	2.38 (0.77–7.29)
Bringing own cooking utensils to MWH					
Not possible	92 (21.5)	12 (5.1)	79 (41.8)	1	1
Possible	333 (77.8)	223 (94.9)	110 (58.2)	13.35 (6.98–25.53)*	0.68 (0.15–3.12)
Stay at MWH 2–4 weeks before delivery					
Not possible	136 (31.8)	9 (3.8)	126 (66.7)	1	1
Possible	289 (67.5)	226 (96.2)	63 (33.3)	50.22 (24.16–104.39)*	18.13 (5.62–58.46)*
Stay attendant at MWH 2–4 weeks before delivery					
Not possible	194 (45.3)	27 (11.5)	166 (87.8)	1	1
Possible	231 (54.0)	208 (88.5)	23 (12.2)	55.60 (30.75–100.54)*	3.33 (1.13–9.83)*
Child care by others while staying at MWH					
Not possible	197 (46.0)	28 (12.0)	168 (89.4)	1	1
Possible	225 (52.6)	205 (88.0)	20 (10.6)	61.50 (33.45–113.07)*	9.33 (2.67–32.65)*
Household care by others while staying at MWH					
Not possible	203 (47.4)	32 (13.7)	170 (89.9)	1	1
Possible	220 (51.4)	201 (86.3)	19 (10.1)	56.20 (30.74–102.74)*	1.73 (0.50–5.93)
Being away from own work					
Not possible	70 (16.4)	18 (7.7)	51 (27.1)	1	1
Possible / no work	355 (82.9)	137 (72.9)	137 (72.9)	4.49 (2.52–8.00)*	1.10 (0.31–3.95)
Attendant being away from work					
Not possible	233 (54.4)	58 (24.7)	174 (92.1)	1	1
Possible	192 (44.9)	177 (75.3)	15 (7.9)	35.40 (19.33–64.83)*	3.77 (1.31–10.86)*

*p < 0.05

## Discussion

This is the first study in Africa that describes associations between determinants and perceived barriers and potential utilization of a Maternity Waiting Home. The results show that MWHs are unknown to 93% of the target population. The most important predictors of intended use are having experienced complications during past deliveries and envisioning relatively few barriers to using an MWH. Barriers that negatively affect a woman’s willingness to use an MWH are being away from the household, leaving her children at home in the care of others, the cost of transport, and the burden on the attendant.

This study was done with limited resources as part of a needs assessment before establishing an MWH and has its limitations. Firstly, only one MWH was available in the Eastern Gurage Zone at the time of the study, which may partially explain why the intervention was unknown to most of the population. Secondly, convenience sampling was applied, which led to underrepresentation of women from rural areas (only 49% of our sample compared to 89% in the Gurage Zone) [7]. The percentage of health facility births is higher compared to the 2016 DHS (39% vs. 26%), which may be the result of data collection in the vicinity of health centres [5]. Otherwise, the socio-demographic profile of our respondents is comparable to the population of Ethiopia [15]. Thirdly, since we did not have the resources to use translators or revisit households of women who fulfilled the inclusion criteria but were not home at the time of our visit, we used a substitution procedure that may have introduced bias. Fourthly, wide confidence intervals around several estimates warrant careful interpretation of these findings. In particular, a wide confidence interval around the odds ratio for “stay at MWH 2-4 weeks before delivery” limits our confidence on the magnitude of the effect size. Despite these shortcomings, we believe that our results provide valuable insight into the level of awareness of our target population, their attitude towards MWHs, and the barriers that women perceive.

It was previously found that a crucial element in the implementation of an MWH is to determine the level of community support, since the success of an MWH depends greatly on acceptance by and support from the community [10, 16–18]. Since most women had never heard of an MWH prior to our study and MWHs were implemented at all health centres shortly after our study was completed, it appears that the level of community support was not determined before implementation. To successfully implement the MWH program in Ethiopia, marginalized women should be heard to determine whether MWHs meet their needs or whether other strategies would be more appropriate to access life-saving emergency obstetric care. In case of sufficient community support, evidence-based interventions should be combined: MWHs alone will not reduce maternal and neonatal mortality and morbidity; they are merely a tool to increase the number of women who are able to access care [19]. A 2015 facility assessment in the Eastern Gurage Zone clearly revealed that more focus is needed on the quality of maternity care, since none of the health centres performed all basic emergency obstetric and newborn care services [20]. If the Ethiopian health care system is incapable of absorbing an influx of women for childbirth, encouraging women to use MWHs could lead to more women receiving substandard care, which may backfire on Ethiopia’s attempts to reduce maternal and neonatal morbidity and mortality.

Our findings indicate that those who suffered complications in previous childbirths were more likely to use an MWH during the following pregnancy. This is in line with several, mostly qualitative, studies, which found that complications during previous births may make women aware of the dangers of childbirth and the benefits of a skilled birth attendant [13, 21, 22]. Many Ethiopians argue that birth is a natural life event that is supposed to take place at home. In their view, a health facility should only come into play when labour is complicated [23, 24]. Since in 20% of low-risk pregnancies complications occur that require treatment, MWHs also target women from remote areas [2]. In the bivariate analyses in this study, however, potential MWH users were more likely to be urban, wealthier, educated women residing at less than 60 min from a hospital, who had (some) decision-making power and had given birth at a health facility in the past. Our findings are similar to a study in Timor-Leste, which found that women living within five kilometres were more likely to use an MWH [18]. Another Ethiopian study studied an MWH that was used by poor, illiterate women living on average 40 km from the hospital. The authors report that its success was rooted in strong community links and acceptance, as well as reliable obstetric services [10]. With facility births at 26% in Ethiopia, reaching out to women with a higher socioeconomic status is not unimportant, but focus should clearly be on those without formal schooling, because of the potential underuse of MWH and facility birth among these women [5].

If Ethiopia is dedicated to maximizing MWH utilization, awareness needs to be raised and barriers need to be overcome, especially among marginalized women. Existing grassroots programs such as the Health Extension Program (community-based health program using locally selected, salaried health workers) and Health Development Army (women-centred community networks) are most likely to reach the target population. Women who suffered pregnancy-related complications in the past and gave birth safely after staying at an MWH could become important local advocates. Aforementioned networks may also be used to co-create solutions as to how child care can best be organized in the community during a woman’s absence and how the burden on the attendant could be relieved. Furthermore, MWH use should be considered when developing a birth plan during antenatal care visits.

Best practices from other projects may be suitable in or could be adapted to the Ethiopian setting. For example, health extension workers and former traditional birth attendants could be trained on which women to refer to the MWH [10]. In Liberia, traditional birth attendants accompany women to an MWH, which led to a substantial increase in facility births [25]. Since ambulances are used for emergencies only, transport vouchers may be given to high-risk and remote pregnant women to reach an MWH free-of-charge [26, 27]. Also, community saving schemes could help raise funds for transport to and from the MWH, while a vegetable garden near the MWH could provide a source of food. We recommend performing research to explore what works best in the Ethiopian setting.

## Conclusions

Most respondents had no knowledge about an MWH. After learning about the concept, willingness to use an MWH was significantly lower among marginalized women, while women who had complications during past births and those who envisioned few barriers expressed that they were more likely to use an MWH in the future. Unless community awareness increases, knowledge of preventive maternity care improves and barriers preventing their use are overcome, MWHs will continue to be underutilized.

## Additional files

Additional file 1: Data collection instrument - English version informed consent form and questionnaire. (PDF 856 kb)

Additional file 2: Data collection instrument – Amharic version informed consent form and questionnaire. (PDF 786 kb)

## Abbreviations

aOR: Adjusted Odds Ratio
CBR: Crude Birth Rate
CI: Confidence Interval
MWH: Maternity Waiting Home
OR: Odds Ratio
