# Supplementary material for: Factors associated with intended use of a maternity waiting home in Southern Ethiopia: a community-based cross-sectional study
Source: BMC Pregnancy Childbirth. 2018 Jan 19;18:38. doi: 10.1186/s12884-018-1670-z (PMC5775531; doi:10.1186/s12884-018-1670-z)
Supplement: Supplementary file 1 — Data collection instrument - English version informed consent form and questionnaire. (PDF 856 kb) [file 12884_2018_1670_MOESM1_ESM.pdf]

Survey number [ ] [ ] [ ] [ ] Data collector ID [ ][ ]

Informed Consent

Survey Maternal Health & Maternity Waiting Home

Introduction & Informed consent	
"Hello. My name is ____ and I am working with an NGO called ***VSO***. We are conducting a research amongst mothers who are currently pregnant or who have delivered in the last three years in the Gurage Zone. The objective is to find out more about maternal health and the use of Maternity Waiting Homes. The information we collect will help Butajira Hospital to improve its maternal health services. You were selected for the survey. The survey usually takes about **45 minutes**. All of the answers you give will be confidential and will not be shared with anyone other than members of our survey team. You don't have to be in the survey, but we hope you will agree to answer the questions since your views are important. If I ask you any question you don't want to answer, just let me know and I will go on to the next question or you can stop the interview at any time.<br><br>Do you have any questions?<br>May I begin the interview now?<br><br>For the interview it is important for me to know if you have given birth in the last 3 years or if you are currently pregnant?<br>IF YES: CONTINUE THE INTERVIEW. IF NO: "Thank you for your time. For this survey, we are specifically interviewing women who have recently giving birth or who are pregnant now."	
**Name of the respondent:**	
**Name of the household head:**	
Signature of interviewer:	Date:
**RESPONDENT AGREES TO BE INTERVIEWED -> Start with question 1**	**RESPONDENT DOES NOT AGREE TO BE INTERVIEWED -> End of interview**

V 25june2014

Survey number [ ] [ ] [ ] [ ] Data collector ID [ ][ ]

Survey Maternal Health & Maternity Waiting Home

1 - Interviewer visit				
**Nr**	**Variable**	**Questions & filters**	**Coding categories**	
1.	Cluster	**Location of interview**<br><br>TO BE FILLED IN BY THE INTERVIEWER	MWH Attat Hospital	1
			Post-labor ward Attat Hospital	2
			MCH Attat Hospital	3
			ANC Attat Hospital	4
			Post-labor ward Butajira	5
			MCH Butajira	6
			ANC Butajira Hospital	7
			Community	8
			Other location, specify:	99
2.		**Data collector ID**<br>TO BE FILLED IN BY THE INTERVIEWER		
3.		**Language interview**<br>TO BE FILLED IN BY THE INTERVIEWER	Amharic	1
			Other, specify:	99
4.		**Language respondent**	Amharic	1
			Other, specify:	99
5.		**Supervisor**<br>TO BE FILLED IN BY THE INTERVIEWER		
6.		**Office editor**<br>TO BE FILLED IN BY OFFICE EDITOR		

2 - Identification				
**Nr**	**Variable**	**Questions & filters**	**Coding categories**	
7.	Household number	**Household number**		
8.	Household head	**Household head**	Male	1
			Female	2
9.	Locality	**Locality Name**		
10.	Woreda	**Woreda**	Butajira City Administration	1
			Meskan	2
			Mareko	3
			Soddo	4
			Other, specify	99
11.	Region	TO BE FILLED IN BY THE INTERVIEWER:<br>Urban / Rural<br><br>Urban: Butajira City, Administration, Attat, Welkite<br>Rural: outside these areas	Urban	1
			Rural	2
12.		TO BE FILLED IN BY OFFICE EDITOR: SURVEY #		

3 - Respondent's background & work

Nr	DHS	Variable	Questions & filters	Coding categories		Skip
	EXPLAIN: I will start the survey with some questions about your background.					
**13.**	102	Age	In what year were you born?	Year		
				Don't know year	98	
**13A.**	103A	Age estimation	IN CASE OF DOUBT: ESTIMATION OF AGE CATEGORY BY THE INTERVIEWER	19 - 24	1	
				25 - 29	2	
				30 - 34	3	
				35 - 39	4	
				40 - 44	5	
				45 - 49	6	
**14.**	603	Marital status	What is your marital status?<br><br>IF MARRIED, SPECIFY IF 'NOT LIVING TOGETHER' AND/OR 'POLYGAMY MARRIAGE'	Single	1	16
				Living together, not married	2	16
				Married	3	
				Not living together	3a	
				Polygamy	3b	
				Separated/divorced	4	
				Widowed	5	
**15.**	611	Age at first marriage	How old were you when you (first) got married?	Age in years		
**16.**	113	Religion	What is your religion?	Orthodox	1	
				Catholic	2	
				Protestant	3	
				Muslim	4	
				Traditional	5	
				Other, specify:	99	
**17.**	114	Ethnicity	What is your ethnicity?	Sebat Bet Gurage	1	
				Silt'e	2	
				Soddo Gurage	3	
				Mareqo / Libido	4	
				Amhara	5	
				Kebena	6	
				Other, specify:	99	
**18.**	104	School attendance	Have you ever attended school?	Yes	1	
				No	2	20
**19.**	105	Educational level	What is the highest level of school you attended: primary, secondary, technical/vocational or higher?	Primary	1	20
				Secondary	2	21
				Technical/vocational	3	21
				Higher	4	21

Survey number [ ] [ ] [ ] [ ]   Data collector ID [ ][ ]

Nr	DHS	Variable	Questions & filters	Coding categories	Skip	
**20.**	108	Literacy	Now I would like you to read this sentence to me<br><br>SHOW SENTENCE TO RESPONDENT.<br>IF RESPONDENT CANNOT READ WHOLE SENTENCE, PROBE: Can you read any part of the sentence to me?<br><br>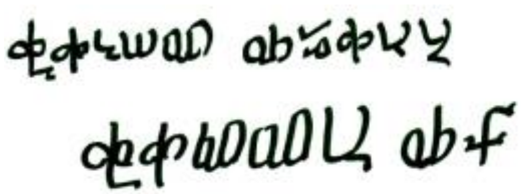	Cannot read at all	1	
				Able to read only parts of the sentence	2	
				Able to read whole sentence	3	
				No card with required language<br>Specify language:	4	
				Blind/visually impaired	5	
**21.**	111	Exposure mass media radio	Do you listen to the radio at least once a week, less than once a week or not at all?	At least once a week	1	
				Less than once a week	2	
				Not at all	3	
**22.**	112	Exposure mass media TV	Do you watch television at least once a week, less than once a week or not at all?	At least once a week	1	
				Less than once a week	2	
				Not at all	3	
**23.**	807	Woman's employment status	Aside from your own housework, have you done any work in the past 12 months?<br><br>IF NECESSARY, EXPLAIN: As you know, some women take up jobs for which they are paid in cash or kind. Others sell things, have a small business or work on the family farm or in the family business.	Yes	1	
				No	2	25
**24.**	811	Woman's occupation	What is your occupation, what kind of work do you mainly do?<br><br>*If the answer to this question is: "housewife", then the answer to 23 is 'No'.*	Merchant	1	
				Farming	2	
				Maid	3	
				Secretary/clerical	4	
				Other, specify:	99	

4 - Partner/Husband's background						
IF ANSWER TO QUESTION 14 IS 1, SKIP TO SECTION 6: HOUSEHOLD WEALTH						
EXPLAIN: Now I would like to ask you some questions about your (late) husband/partner's background and the way the household is run.						
**Nr**	**DHS**	**Variable**	**Questions & filters**	**Coding categories**		**Skip**
**25.**	803	Partner's educational level	Did your (last) husband/partner ever attend school?	Yes	1	
				No	2	27
**26.**	804	Partner's educational level	What is the highest level of school your husband attended: primary, secondary, technical/vocational or higher?	Primary	1	
				Secondary	2	
				Technical/vocational	3	
				Higher	4	
**27.**	806	Partner's employment status	What was your (last) husband's/ partner's occupation? That is, what kind of work does/did he mainly do?	Merchant	1	
				Farming	2	
				Driver	3	
				Government employee	4	
				Unemployed	5	
				Other, specify:	99	

5 - Household chores / decision-making						
**Nr**	**DHS**	**Variable**	**Questions & filters**	**Coding categories**		**Skip**
**28.**		Decision-making earnings	Who usually decides how the family earnings are spent?	Respondent	1	
				Husband/partner	2	
				Jointly	3	
				No earnings	4	
				Other, specify:	99	
**29.**	822A	Division household chores	Does your husband help you with household chores like looking after the children, cooking, cleaning the house, and doing other work around the house?	Yes	1	
				No	2	
**30.**	825	Presence others	PRESENCE OF OTHERS AT THIS POINT<br>1. PRESENT AND LISTENING<br>2. PRESENT BUT NOT LISTENING<br>3. NOT PRESENT	CHILDREN < 10	1<br>2<br>3	
				HUSBAND	1<br>2<br>3	
				OTHER MALES	1<br>2<br>3	
			INTRODUCE THE NEXT QUESTION: In some parts of Ethiopia, husbands hit or beat their wife.	OTHER FEMALES	1<br>2<br>3	
**31.**	826	Domestic/ sexual violence	In your opinion, is a husband justified in hitting or beating his wife?	Yes	1	
				No	2	

Survey number [ ] [ ] [ ] [ ]　　　　Data collector ID [ ][ ]

6 - Household wealth						
**Nr**	**DHS**	**Variable**	**Questions & filters**	**Coding categories**		**Skip**
**32.**		Household wealth	Does your household have/own…			
			• Food sufficiency	Yes	1	
				No	2	
			• Running water	Yes	1	
				No	2	
			• Electricity	Yes	1	
				No	2	
			• Television	Yes	1	
				No	2	
			• Mobile phone	Yes	1	
				No	2	
			• Land	Yes	1	
				No	2	
			• Livestock	Yes	1	
				No	2	
**33.**		Relative household wealth	How wealthy is your household compared to other households in your community?	Very wealthy	1	
				Wealthy	2	
				Poor	3	
				Very poor	4	

	7 - Reproduction & Contraception					
	Now I would like to ask about all the births you have had during your life.					
**Nr**	**DHS**	**Variable**	**Questions & filters**	**Coding categories**		**Skip**
**34.**	201	Birth giving	Have you ever given birth?	Yes	1	
				No	2	36
**35.**	…	Total number of live births	How often did you give birth to a live baby?			
**36.**	…	IUFD	Have you ever given birth to a boy or girl who was dead?	Yes	1	
				No	2	38
**37.**	…	Total number of IUFD	How often did you give birth to a dead baby?			
**38.**	206	Early neonatal death	Have you ever given birth to a boy or girl who was born alive but died within the first week?<br><br>IF NO, PROBE:<br>Any baby who cried or showed signs of life but did not survive?	Yes	1	
				No	2	40
**39.**		Total number of early neonatal deaths	How often have you given birth to a boy or girl who was born alive but died within a week?			
**40.**	208	Total number of births	TOTAL NUMBER OF BIRTHS, INCL STILLBIRTHS.<br>CALCULATE: ANSWER QUESTION 35 + 37<br>IF NONE, RECORD '00'.			

**41.**	226	Pregnancy	Are you pregnant now?	Yes	1	
				No	2	45
				Unsure	98	45
IF THE ANSWER TO QUESTION 40 IS "00" (ZERO) AND THE ANSWER TO QUESTION 41 IS "NO", CHECK WITH THE RESPONDENT IF THIS IS CORRECT. IF THE ANSWERS REMAIN UNCHANGED, STOP THE INTERVIEW HERE.						
**42.**	227	Completed months of pregnancy	How many months pregnant are you?<br><br>RECORD NUMBER OF COMPLETED MONTHS	Months……		
**42a**		ANC Visits	How many ANC visits did you have?			
**42b**		Place ANC	Where did you have ANC?	Health Post	1	
				Health Center	2	
				Hospital	3	
				Other, specify:	99	
**43.**		Knowledge EDD	Do you know your expected delivery date?	Yes	1	
				No	2	
**44.**	228	(Un)wanted/ planned/supported pregnancy	Is the pregnancy planned, wanted, supported?<br><br>RECORD ALL	Planned	1	
				Wanted	2	
				Supported	3	
				None of the above	4	
**45.**	313	Contraceptive use	Have you ever used anything or tried in any way to delay or avoid getting pregnant?	Currently using	1	
				Used in the past	2	
				Never used	3	
**46.**	820	Decision-making MCH	Who usually makes decisions about maternal & child health care for yourself and your baby?	Respondent	1	
				Husband/partner	2	
				Jointly	3	
				Other, specify:	99	

Survey number [ ] [ ] [ ] [ ]   Data collector ID [ ][ ]

7 continued - Reproduction – Births

RECORD ALL BIRTHS (INCL STILLBIRTHS). FOR A MULTIPLE PREGNANCY, USE 1 COLOMN TO RECORD 1 CHILD. IF THERE ARE MORE THAN 6 CHILDREN, USE AN EXTRA SURVEY.									SKIP
**Nr**	**Variables**	**Filters**	**1st birth**	**2nd birth**	**3rd birth**	**4th birth**	**5th birth**	**6th birth**	
**47.**	Name of child								
**48.**	Year of birth								
**49.**	Is the child still alive?	Yes	1	1	1	1	1	1	**51**
		No	2	2	2	2	2	2	
**50.**	At what age did the child die?	Age in completed min/days/ weeks/ months/years							
**51.**	How old is the child now?	Age in completed years							
**52.**	Number of ANC Visits								
**53.**	Place of delivery	Home	1	1	1	1	1	1	
		Health facility	2	2	2	2	2	2	
IF ALL DELIVERIES WERE IN THE SAME PLACE, FILL IN THE FOLLOWING QUESTIONS FOR THE MOST RECENT DELIVERY. IF THERE IS A CHANGE IN PLACE OF DELIVERY (HOME TO HF, OR HF TO HOME), FILL IN THE FOLLOWING QUESTIONS FOR THESE DELIVERIES. FACILITY DELIVERY: HOME DELIVERY:									**54** **55**
**54.**	Why did you deliver in a health facility? PROBE AND RECORD ALL REASONS	Comfortable	1	1	1	1	1	1	**57**
		Privacy	2	2	2	2	2	2	
		Complications in previous pregnancies/deliveries	3	3	3	3	3	3	
		Complications during labor	4	4	4	4	4	4	
		Husband/family decided	5	5	5	5	5	5	
		No/low costs	6	6	6	6	6	6	
		Other, specify	99	99	99	99	99	99	

	Variables	Filters	1st birth	2nd birth	3rd birth	4th birth	5th birth	6th birth	SKIP
**55.**	Why did you deliver at home?<br><br>PROBE AND RECORD ALL REASONS.	Comfortable	1	1	1	1	1	1	
		Privacy	2	2	2	2	2	2	
		Cultural rituals (presence TBA, placenta burial, massage, etc.)	3	3	3	3	3	3	
		Husband/family decided	4	4	4	4	4	4	
		No complications	5	5	5	5	5	5	
		No/low costs	6	6	6	6	6	6	
		Other, specify	99	99	99	99	99	99	
**56.**	Why didn't you deliver in a health facility?<br><br>PROBE AND RECORD ALL REASONS.	Cost too much	A	A	A	A	A	A	
		Facility (often) not open / possibility of onwards referral	B	B	B	B	B	B	
		Too far/no transportation	C	C	C	C	C	C	
		Don't trust facility	D1	D1	D1	D1	D1	D1	
		Poor quality of service	D2	D2	D2	D2	D2	D2	
		No female provider at facility	E	E	E	E	E	E	
		Husband/family did not allow	F	F	F	F	F	F	
		No family members allowed	G	G	G	G	G	G	
		No privacy	H	H	H	H	H	H	
		No cultural rituals	I	I	I	I	I	I	
		No complications	J	J	J	J	J	J	
		Fear of an operation	K	K	K	K	K	K	
		Other, namely:	99	99	99	99	99	99	
**57.**	Mode of delivery	SVD	1	1	1	1	1	1	
		VE	2	2	2	2	2	2	
		CS	3	3	3	3	3	3	

Survey number [ ] [ ] [ ] [ ]   Data collector ID [ ][ ]

	Variables	Filters	1st birth	2nd birth	3rd birth	4th birth	5th birth	6th birth	SKIP
**58.**	Complications	Yes	1	1	1	1	1	1	
		No	2	2	2	2	2	2	**63**
**59.**	Which complications?	Hemorrhage	1	1	1	1	1	1	
		Prolonged labor	2	2	2	2	2	2	
	PROBE AND RECORD ALL REASONS.	Obstructed labor	3	3	3	3	3	3	
		Hypertensive disorder	4	4	4	4	4	4	
		Puerperal infection	5	5	5	5	5	5	
		Fetal distress baby	6	6	6	6	6	6	
		IUFD	7	7	7	7	7	7	
		Other, namely:	99	99	99	99	99	99	
								IF IT WAS A FACILITY DELIVERY:	**63**
**60.**	Did you seek medical advice or treatment for these complications?	Yes	1	1	1	1	1	1	**61**
		No	2	2	2	2	2	2	**62**
**61.**	Where did you seek medical advice or treatment for these complications?	TBA	1	1	1	1	1	1	**63**
		Health facility	2	2	2	2	2	2	
		Other, namely:	99	99	99	99	99	99	
**62.**	Why didn't you seek medical advice or treatment for these complications?	Cost too much	A	A	A	A	A	A	
		Facility (often) not open / possibility of onwards referral	B	B	B	B	B	B	
		Too far/no transportation	C	C	C	C	C	C	
	PROBE AND RECORD ALL REASONS	Don't trust facility	D1	D1	D1	D1	D1	D1	
		Poor quality of service	D2	D2	D2	D2	D2	D2	
		No female provider at facility	E	E	E	E	E	E	
		Husband/family did not allow	F	F	F	F	F	F	
		Not necessary	G	G	G	G	G	G	
		Not customary	H	H	H	H	H	H	
		Other, namely:	99						

Survey number [ ] [ ] [ ] [ ] Data collector ID [ ][ ]

8 - KNOWLEDGE OF PREGNANCY COMPLICATIONS & BIRTH PREPAREDNESS						
IF THE RESPONDENT NEVER HAD ANC, SKIP TO						**64**
**Nr**	**DHS**	**Variable**	**Questions & filters**	**Coding categories**		**Skip**
**63.**	414	Health education complications	During any of your ANC visits, where you told the signs of pregnancy complications?<br><br>IF UNCLEAR, EXPLAIN: Complications of pregnancy are health problems that occur during pregnancy. They can involve the mother's health, the baby's health, or both.	Yes	1	
				No	2	
**64.**	414A	Knowledge pregnancy-complications	Can you tell me any signs of pregnancy complications?<br><br>GIVE THE RESPONDENT TIME TO THINK FIRST, THEN PROBE. RECORD ALL ANSWERS.	Vaginal bleeding	A	
				Vaginal flush of fluid	B	
				Severe headache	C	
				Blurred vision	D	
				Fever	E	
				Abdominal pain / preterm contractions	F	
				Decreased fetal movement	G	
				Edema/body swelling	H	
				Other, specify:	99	
IF THE RESPONDENT DOESN'T KNOW SIGNS OF PREGNANCY COMPLICATIONS, PLEASE NAME THEM BEFORE CONTINUING WITH THE NEXT QUESTION						
IF THE RESPONDENT NEVER HAD ANC, SKIP TO						**66**
**65.**		Health education birth preparedness	During any of your ANC visits, where you advised about birth preparedness?<br><br>IF THE RESPONDENT DOESN'T KNOW WHAT IT MEANS, EXPLAIN THE CONCEPT.	Yes	1	
				No	2	
**66.**		BP: Advance planning place of delivery	During your pregnancies, did you plan in advance where you wanted to give birth?	Yes	1	
				No	2	73
**67.**		BP: planned place of delivery	Where did you plan to give birth?	Home	1	68
				Health facility	2	69
				Other, namely	99	
**68.**		BP: Trained BA	Did you identify a trained birth attendant?	Yes	1	
				No	2	
**69.**		BP: HF	Did you identify a health facility (for an obstetric emergency)?	Yes	1	
				No	2	
**70.**		BP transport	Did you identify a mode of transport to the facility (for obstetric emergency)?	Yes	1	
				No	2	

**71.**		BP: money	Did you save money for the delivery and/or an emergency?	Yes	1	
				No	2	
**72.**		BP: blood donor	Did you identify a blood donor?<br><br>EXPLAIN: in case of an emergency, you may need blood from a donor, which should be the same as your own blood type (e.g. A+, B or O)	Yes	1	
				No	2	

9 - Perceived risk

Nr	DHS	Variable	Questions & filters	Coding categories		Skip
**73.**		Perceived risk mother	Where do believe that you run the greatest risk during a delivery, at home or in a health facility?	Home	1	
				Health facility	2	
**74.**		Perceived risk - baby	Where do you believe that your baby runs the greatest risk during a delivery, at home or in a health facility?	Home	1	
				Health facility	2	

10 - Health Services & Community Context

EXPLAIN: Now I would like to ask about all the health services in your community and about your experiences with those services

Nr	DHS	Variable	Questions & filters	Coding categories		Skip
**75.**		Travel time Health Post	What is the travel time from your household to the nearest Health Post?<br><br>RECORD THE TOTAL TIME IN MINUTES AND/OR HOURS	Minutes		
				Hours		
				No HP	98	
**76.**		Travel time Health Center	What is the travel time from your household to the nearest Health Center?	Minutes		
				Hours		
				No HC	98	
**77.**		Travel time Hospital	What is the travel time from your household to the nearest Hospital?	Minutes		
				Hours		
**78.**		Emergency transport	Once labor has started at home, how easy or difficult is it to find transport?	Very easy	1	
				Easy	2	
				Difficult	3	
				Very difficult	4	
**79.**		Emergency mode of transport	In case of an emergency during home delivery, what mode of transport is used for the majority of the journey to the nearest hospital?	Walking / Carried	1	
				Horse	2	
				Public transport	3	
				Private transport	4	
				Ambulance	5	

Survey number [ ] [ ] [ ] [ ]   Data collector ID [ ][ ]

11 - Perceived quality of maternal & child services						
**Nr**	**DHS**	**Variable**	**Questions & filters**	**Coding categories**		**Skip**
**80.**		Nearest hospital	Which hospital is closest to your home?	Attat Hospital (NGO)	A	
				Butajira Hospital (Govt)	B	
				Mercy Hospital (NGO)	M	
				Other, specify:	99	
**81.**		Utilization hospital	Did you ever go to this hospital?	Yes	1	
				No	2	**84**
**82.**		Utilization MCH at hospital	Have you ever been to this hospital for maternal & child health services (ANC, ultrasound, delivery care, post-natal care)?	Yes	1	
				No	2	**84**
**83.**		Utilization types of MCH services at hospital	Which maternal & childcare services did you use at the hospital?<br><br>ANC: medical care for pregnant women during their pregnancy.<br>Ultrasound: a machine that allows to see how a baby is developing in the womb. Gel is put on your belly and a probe moves around, which creates a picture of your baby on the machine.<br>Delivery care: giving birth with a skilled birth attendant.<br>Post-natal care: care to mother and child after the baby is born.<br><br>RECORD ALL SERVICES USED	ANC	A	**85**
				Ultrasound	B	
				Delivery care	C	
				Post-natal care	D	
**84.**		Reasons for non-utilization (MCH at) hospital	What is your reason for not going to the hospital (for MCH services)?<br><br>RECORD ALL REASONS.	Cost too much	A	**97**
				Facility not open	B	
				Too far/no transportation	C	
				Don't trust facility	D1	
				Poor quality of service	D2	
				No female provider at facility	E	
				Husband/family did not allow	F	
				Not necessary	G	
				Not customary	H	
				Other, specify	99	

Survey number [ ] [ ] [ ] [ ]   Data collector ID [ ][ ]

			How do you perceive the quality of maternal & childcare at the hospital with regard to the following services? Please be frank, as it will help us improve our services to you and other patients.			
**85.**		Perceived quality of care MCH hospital - overall	• Overall service	Good	4	
				Satisfactory	3	
				Reasonable	2	
				Poor	1	
**86.**		Perceived quality of care MCH hospital - supplies	• Availability of supplies: drugs, blood, other medical materials	Good	4	
				Satisfactory	3	
				Reasonable	2	
				Poor	1	
**87.**		Perceived quality of care MCH hospital – waiting times	• Waiting times	Good	4	
				Satisfactory	3	
				Reasonable	2	
				Poor	1	
**88.**		Perceived quality of care MCH hospital – quality staff	• Professionalism of staff	Good	4	
				Satisfactory	3	
				Reasonable	2	
				Poor	1	
**89.**		Perceived quality of care MCH hospital – friendliness staff	• Friendliness of staff	Good	4	
				Satisfactory	3	
				Reasonable	2	
				Poor	1	
**90.**		Perceived quality of care MCH hospital – respect preferences	• Respect for preferences	Good	4	
				Satisfactory	3	
				Reasonable	2	
				Poor	1	
**91.**		Perceived quality of care MCH hospital – privacy	• Privacy	Good	4	
				Satisfactory	3	
				Reasonable	2	
				Poor	1	
**92.**		Perceived quality of care MCH hospital - hygiene	• Hygiene	Good	4	
				Satisfactory	3	
				Reasonable	2	
				Poor	1	
**93.**		Perceived quality of care MCH hospital - language	• Own language spoken	Yes	1	
				No	2	
**94.**		Perceived quality of care MCH hospital - discriminatio n	• Discrimination against certain groups<br><br>EXPLAIN: Sometimes, some people discriminate against others based on ethnicity, living standard and/or diseases like HIV.	Yes	1	
				No	2	
**95.**		Perceived	• Cost	Affordable	1	

		quality of care MCH hospital - cost		Not affordable	2	
**96.**		Recommendation MCH Hospital	Would you recommend the MCH services to other pregnant women?	Yes	1	
				No	2	

**12 A - MWH for non-MWH women**						
**Nr**	**DHS**	**Variable**	**Questions & filters**	**Coding categories**		**Skip**
**97.**		Knowledge MWH	Have you ever heard of a Maternity Waiting Home?	Yes	1	
				No	2	101
NOW YOU ALWAYS EXPLAIN/COMPLETE THE CONCEPT OF A MWH: A Maternity Waiting Home is a place for high-risk pregnant women to await birth in their last weeks of pregnancy, close to 24/7 emergency obstetric care. Possible reasons to stay are for example a previous caesarean section or hemorrhage, previous stillbirth or neonatal death, breech presentation, twin pregnancy, or living far from a hospital.						
**98.**		Knowledge MWH: availability	Is a MWH available in your region?	Yes	1	
				No	2	**101**
**99.**		Location MWH	Where is the MWH located?	Attat Hospital	A	
				Mercy Hospital	M	
				Other, specify:	99	
**100.**		Stay MWH	Did you ever stay at a Maternity Waiting Home?	Yes	1	**116**
				No	2	
**101.**		Knowledge MWH: advantages	What do you think the advantages are of staying at a MWH?<br><br>RECORD ALL ANSWERS.	Closeness to emergency obstetrics care	1	
				Saving life of mother	2	
				Saving life of baby	3	
				Calmness, rest before delivery	4	
				Other, specify	99	
				Don't know	98	
IF THE RESPONDENT CANNOT THINK OF ANY ADVANTAGES, EXPLAIN THE ADVANTAGES.						
**102.**		Likeliness stay MWH	For your current/next pregnancy, how likely is it that you will stay at a MWH the last 2-4 weeks prior to your delivery?	Very likely	1	
				Likely	2	
				Somewhat unlikely	3	
				Very unlikely	4	
	EXPLAIN: Now imagine you have to stay at the MWH for 2-4 weeks prior to your expected delivery date. How will it be for you to make the following arrangements?					
**103.**		Barrier MWH transport	• Transport to and from MWH	Affordable	1	
				Not affordable	2	
**104.**		Barrier MWH food	• Food while staying at MWH	Affordable	1	
				Not affordable	2	
**105.**		Barrier MWH utensils	• Bringing own cooking utensils	Possible	1	
				Impossible	2	
**106.**		Barrier MWH	• Staying for 2-4 weeks before	Possible	1	

		waiting time respondent	delivery date	Not possible	2	
**107.**		Barrier MWH waiting time attendant	• Bringing an attendant for at least 2 weeks before delivery date	Possible	1	
				Not possible	2	
**108.**		Barrier MWH children at home	• Children are taken care of by other family members / community	Possible	1	
				Not possible	2	
**109.**		Barrier MWH household chores	• Household chores are taken care of by family / community	Possible	1	
				Not possible	2	
**110.**		Barrier MWH loss from productivity activity	• Being away from your work (other than household chores)	Possible	1	
				Not possible	2	
**111.**		Barrier MWH attendant loss productivity	• Attendant being away from other work / obligations	Possible	1	
				Not possible	2	
**112.**		Barrier MWH dependent child	• Bringing a child to MWH	Necessary	1	
				Not necessary	2	
**113.**		Barrier MWH family visits	• Visits from family members	Necessary	1	
				Not necessary	2	
**114.**		Other barriers MWH	Are there any other challenges to staying at a MWH?	Yes	1	
				No	2	End of survey
**115.**		Other barriers specified	If yes, please explain what these challenges are.		99	End of survey

**12 B - MWH for MWH-women**						
**Nr**	**DHS**	**Variable**	**Questions & filters**	**Coding categories**		**Skip**
**116.**		Location stay MWH	Which MWH did you stay?	Attat Hospital	A	
				Mercy Hospital	M	
				Other, specify:	99	
**117.**		Reason admission MWH	What was the reason for your admission to the MWH?<br><br>IF THE RESPONDENT DOES NOT KNOW THE ANSWER: ASK YOUR SUPERVISOR TO CHECK THE MEDICAL FILE AND THEN RECORD THE ANSWER(S).	Previous caesarean section	1	
				Previous obstetric fistula repair	2	
				Multiple pregnancy	3	
				Grand multiparity	4	
				Previous stillbirth/neonatal loss	5	
				Malpresentations	6	
				Breech presentation	7	
				Antepartum hemorrhage	8	
				Anemia	9	
				Polyhydramnios	10	

				Preeclampsia/medical problems	11	
				Living far away from a hospital with emergency obstetrics care	12	
				Other, specify:	99	
**118.**		Referral to MWH	From where were you referred to the MWH?	HEW	1	
				HP	2	
				HC	3	
				Hospital	4	
				Other, specify:	99	
**119.**		Duration stay MWH	How long did you stay in the MWH?<br><br>RECORD THE NUMER OF WEEKS IN HALF AND/OR WHOLE NUMBERS.<br><br>IF THE RESPONDENT IS STILL STAYING AT THE MWH, RECORD THE LENGTH OF STAY UNTIL NOW.	Length of stay		
**120.**		Decision-making MWH	Who made the decision to stay at the MWH?	Respondent	1	
				Husband/partner	2	
				Jointly	3	
				Other, specify	99	
**121.**		Attendant MWH	Who was your attendant during your stay at the MWH?	Husband/partner	1	
				Other family member	2	
				Other, specify:	99	
**122.**		Financial support MWH	Who provided financial support during your stay at the MWH? (Transport, food, medications)	Respondent	1	
				Husband	2	
				Other family member	3	
				Other, specify	99	
**123.**		Social support MWH	Who provided social support during your stay at the MWH? (Taking care of children, household chores and/or other work, emotional support)	Husband	1	
				Other family member	2	
				Other, specify	99	
**124.**		Advantages MWH	What are the advantages of staying at a MWH?<br><br>RECORD ALL REASONS MENTIONED.	Closeness to emergency obstetrics care	1	
				Saving life of mother	2	
				Saving life of baby	3	
				Calmness, rest before delivery	4	
				Other, specify	99	

Survey number [ ] [ ] [ ] [ ]   Data collector ID [ ][ ]

			EXPLAIN: I would appreciate if you gave us your opinion about the following items. Please be frank with me, as it will allow the hospital to improve its services.			
**125.**		Barrier MWH transport	• Transport to and from MWH	Affordable	1	
				Not affordable	2	
**126.**		Barrier MWH food	• Food while staying at MWH	Affordable	1	
				Not affordable	2	
**127.**		Barrier MWH utensils	• Bringing own cooking utensils	Possible	1	
				Not possible	2	
**128.**		Barrier MWH waiting time respondent	• Staying for 2-4 weeks before delivery date	Possible	1	
				Not possible	2	
**129.**		Barrier MWH waiting time attendant	• Bringing an attendant for at least 2 weeks before delivery date	Possible	1	
				Not possible	2	
**130.**		Barrier MWH children at home	• Children are taken care of by other family members / community	Possible	1	
				Not possible	2	
**131.**		Barrier MWH household chores	• Household chores are taken care of by family / community	Possible	1	
				Not possible	2	
**132.**		Barrier MWH loss from productivity activity	• Being away from your work (other than household chores)	Possible	1	
				Not possible	2	
**133.**		Barrier MWH attendant loss productivity	• Attendant being away from other work / obligations	Possible	1	
				Not possible	2	
**134.**		Barrier MWH dependent child	• Bringing a child to MWH	Necessary	1	
				Not necessary	2	
**135.**		Barrier MWH family visits	• Visits from family members	Necessary	1	
				Not necessary	2	
**136.**		Other barriers MWH	Are there any other challenges to staying at the MWH?	Yes	1	
				No	2	
**137.**		Other barriers MWH specified	If yes, please explain what these challenges are.		99	
			How do you perceive the quality of care at the MWH with regard to the following services? Please be frank as it will help us improve our services to you and other patients			
**138.**		Perceived quality MWH facilities	• Facilities: accommodation, equipment, supplies	Good	4	
				Satisfactory	3	
				Reasonable	2	
				Poor	1	
**139.**		Quality MWH hygiene	• Hygiene	Good	4	
				Satisfactory	3	
				Reasonable	2	
				Poor	1	
**140.**		Perceived quality MWH privacy	• Privacy	Good	4	
				Satisfactory	3	
				Reasonable	2	

				Poor	1	
**141.**		Perceived quality MWH support women	• Support from other MWH women	Good	4	
				Satisfactory	3	
				Reasonable	2	
				Poor	1	
**142.**		Perceived quaiity MWH space attendants	• Space for attendants / visitors	Good	4	
				Satisfactory	3	
				Reasonable	2	
				Poor	1	
**143.**		MWH areas improvement	What are areas for improvement of the MWH?		99	
**144.**		Recommendation MWH	Would you recommend the MWH to other pregnant women?	Yes	1	End of survey
				No	2	
