# Supplementary material for: Factors associated with intended use of a maternity waiting home in Southern Ethiopia: a community-based cross-sectional study
Source: BMC Pregnancy Childbirth. 2018 Jan 19;18:38. doi: 10.1186/s12884-018-1670-z (PMC5775531; doi:10.1186/s12884-018-1670-z)
Supplement: Supplementary file 2 — Data collection instrument – Amharic version informed consent form and questionnaire. (PDF 786 kb) [file 12884_2018_1670_MOESM2_ESM.pdf]

Survey number [ ] [ ] [ ] [ ]   Data collector ID [ ] [ ]

<u>በማወቅ የሚሰጥ የስምምነት ቅጽ</u>

<u>የእናቶችን ጤና እና የነፈሰጡር እናቶች ማረፊያ ቤትን የተመለከተ ጥናት</u>

መግቢያ እና በማወቅ የሚሰጥ የስምምነት ቅጽ	
"ጤና ይስጥልኝ አቶ/ወሮ_______________________________________እባላለሁ VSO ከሚባል ድርጅት ጋር ነው የምሰራው። በጉራጌ ዞን አሁን ነፍሰጡር የሆኑ እና ባለፉት ሶስት አመታት የወለዱ እናቶችን የሚያካትት ጥናት እያደረግን ነው።የጥናቱ አላማ በዞኑ ያለውን የእናቶችን ጤና እና የነፈሰጡር እናቶች ማረፊያ ቤትን አጠቃቀም በበለጠ ለመረዳት ነው።እርሶ በጥናቱ ላይ እንዲሳተፉ የተመረጡ በመሆኑ እርስዎ የሚሰጡን መረጃ ቡታጅራ ሆስፒታል ለሚሰጠው የእናቶች ጤና አገልግሎት መሻሻል ትልቅ አስተዎፆ ያደርጋል።ፈቃደኛ ከሆኑ ጥያቄዎቹን ለመመለስ ....ደቂቃ ያክል ይወስዳለ የሚሰጡንን መረጃ በሚሰጥር እንደምንይዝ ቃል እየገባንልዎት መመለስ የማይፈልጉት ጥያቄ ካለ አለመመለስ ይችላሉ ወደ ሚቀጥለው ጥያቄ እንሄዳለን።<br>ጥያቄ አለዎት?<br>ጥያቄ ከሌለዎት መጠይቁን መጀመር እንችላለን?<br><br>**መጠይቁን ለመጀመር አሁን ነፈሰጡ መሆንዎን ወይም ባለፉት ሶስት አመት መውለድዎን ማወቅ አለብን ።**<br>**አዎ አሁን ነፈሰጡ ነኝ............አዎ ባለፉት ሶስት አመታት ወልጃለሁ................:መጠይቁን ቀጥይ/ል።**<br>**አይደለሁም ከሆነ "ይቅርታ በጥናቱ ላይ መሳተፍ የሚችሉት አሁን ነፈሰጡር የሆኑ ወይም ባለፉት ሶስት አመታት ውስጥ የወለዱ ናቸው"**	
የተጠያቂዋ ስም:	
የቤተሰቡ ሃላፊ ስም :	
የጠያቂው ፊርማ:	ቀን:
ተጠያቂዋ ከተስማማች -> ወደ ጥያቄ ቁጥር አንድ ሂድ	ተጠያቂዋ ካልተስማማች -> መጠይቁን አቆርጥ

Survey number [ ] [ ] [ ] [ ] Data collector ID [ ] [ ]

የእናቶችን ጤና እና የነፈሰጡር እናቶች ማረፊያ ቤትን የተመለከተ ጥናት

1 - የጠያቂው ጉብኝት				
**Nr**	**Variable**	**Questions & filters**	**Coding categories**	
**1.**	Cluster	*የተጠየቁበት ቦታ*<br><br>(በጠያቂዋ የሚሞላ)	የነፍሰጡር እናቶች ማረፊያ ቤት አጣጥ ሆ/ታ	1
			ደህረ ወሊድ ምኝታ ክፍል አጣጥ ሆ/ታ	2
			የእናቶች እና ህፃናት ጤና አ/ገት አጣጥ ሆ/ታ	3
			ቅድመ ወሊድ ክትትል ክፍል አጣጥ ሆ/ታ	4
			ደህረ ወሊድ ምኝታ ክፍል ቡታጅራ ሆ/ታ	5
			የእናቶች እና ህፃናት ጤና አ/ገት ቡታጅራ ሆ/ታ	6
			ቅድመ ወሊድ ክትትል ክፍል ቡታጅራ ሆ/ታ	7
			የሕብረተሰቡ መኖሪያ አካባቢ	8
			ሌላ አካባቢ : ግለፅ	99
**2.**	**Datacollector ID**	የጠያቂዋ ስም<br>(በጠያቂዋ የሚሞላ)		
**3.**	**Language interview**	የመጠይቁ ቋንቋ<br>(በጠያቂዋ የሚሞላ)	አማርኛ	1
			ሌላ: ግለፅ	99
**4.**	**Language respondent**	የመላሽዋ ቋንቋ	አማርኛ	1
			ሌላ: ግለፅ	99
**5.**		Supervisor<br>(በጠያቂዋ የሚሞላ)		
**6.**		Office editor<br>TO BE FILLED IN BY OFFICE EDITOR		

2 - IDENTIFICATION				
**Nr**	**Variable**	**Questions & filters**	**Coding categories**	
**7.**	Household number	የቤት ቁጥር		
**8.**	Household head	የቤተሰቡ ሃላፊ	ወንድ	1
			ሴት	2
**9.**	Locality	የአካባቢው መጠሪያ		
**10.**	Woreda	ወረዳ	ቡታጅራ ከተማ አስተዳደር	1
			መስቃን	2
			ማረቆ	3
			ሶዶ	4
			ሌላ አካባቢ : ግለፅ	99
**11.**	Region	በጠያቂው/ዋ የሚሞላ:<br>ከተማ/ ገጠር<br>ከተማ:ቡታጅራ ከተማ አስተዳደር<br>ገጠር: ሌላ አካባቢ	ከተማ	1
			ገጠር	2
**12.**		TO BE FILLED IN BY OFFICE EDITOR: SURVEY NUMBER		

3 - መሠረታዊ መርጃ እና ስራ					
አስረጅ፤ጥያቂዎችን እመጀምረው አንዳንድ መሰረታዊ ነገሮችን ስለአንች በመጠየቅ ይሆናል					
**Nr**	**Variable**	**Questions & filters**	**Coding categories**		**Skip**
**13.**	Age	የትውልድ ዘመን/እድሜ?	አመተ ምህረት		
			አላውቀውም	98	
**13A.**	Age estimation	ጥርጣሬ ካለ እባክዎን ይገምቱ፡(ለጠያቂው)	19 - 24	1	
			25 - 29	2	
			30 - 34	3	
			35 - 39	4	
			40 - 44	5	
			45 - 49	6	
**14.**	Marital status	የትዳር ሁኔታ ?<br><br>ያገቡ ሆነው አብረው እየኖሩ ካልሆነ ወይም ከአንድ በላይ ጋብቻ እባክዎን ይግለፁ	ያላገባ	1	16
			አብሮ የሚኖር ያልተጋባ	2	16
			ያገባ	3	
			• አብሮ የማይኖር	3a	
			• ከአንድ በላይ ጋብቻ	3b	
			የተፋታ/የተለያዩ	4	
			ባለቤትዋ የሞተባት	5	
**15.**	Age at first marriage	የመጀመሪያ ሲያገቡ/ትዳር ስትይዥ እድሜዎት ስንት ነበር?	እድሜ		
**16.**	Religion	ሃይማኖትዎ ምንድን ነው?	ኦርቶዶክስ	1	
			ካቶሊክ	2	
			ፕሮቴስታንት	3	
			ሙስሊም	4	
			ባህላዊ ሃይማኖት	5	
			ሌላ : ግለፅ	99	
**17.**	Ethnicity	ብሔርዎ ምንድን ነው?	ሰባት ቤት ጉራጌ	1	
			ስልጢ	2	
			ሶዶ ጉራጌ	3	
			ማረቆ/ሊቢዶ	4	
			ቀቤና	5	
			አማራ	6	
			ሌላ : ግለፅ	99	
**18.**	School attendance	ትምህርት ተምረዋል?	ተምሪያለሁ	1	
			አልተማርኩም	2	20
**19.**	Educational level	የትምህርት ደረጃ?	የመጀመሪያ ደረጃ	1	20
			ሁለተኛ ደረጃ	2	21
			ቴክኒክና ሙያ	3	21
			ከፍተኛ ደረጃ ትምህርት	4	21

Nr	Variable	Questions & filters	Coding categories		Skip
20.	Literacy	እባክዎን ቀጣዩን አረፍተ ነገር ያንቡልኝ<br><br>አረፍተ ነገሩን ያሳዩ።<br><br>መላሽዋ ሙሉውን አረፍተ ነገር ማንበብ ካልቻሉ ፤ ከአረፍተ ነገሩ ውስጥ ሊያነቡት የሚችሉት ቃል ካለ ይጠይቁዋቸው?<br><br>ታሞ ከመማቀቅ<br>አስቀድሞ መጠንቀቅ	ማንበብ አይችሉም	1	
			የተወሰነ ቃላት ያነባሉ	2	
			ሙሉ አ/ነገር ማንበብ ይችላሉ	3	
			ከአማረኛ ቋንቋ ውጭ ማንበብ ይችላሉ<br>ቋንቋውን ግለጽ :	4	
			ማየት የተሳናት/ቸው	5	
21.	Exposure mass media radio	ሬድዮ ምን ያክል ይከታተላሉ?	በሳምንት አንድ ጊዜ እና ከዚያ በላይ	1	
			በሳምንት አንድ ጊዜ እና ከዚያ በታች	2	
			አልከታተልም	3	
22.	Exposure mass media tv	ቴሌቭዥንን ምን ያክል ይከታተላሉ?	በሳምንት አንድ ጊዜ እና ከዚያ በላይ	1	
			በሳምንት አንድ ጊዜ እና ከዚያ በታች	2	
			አልከታተልም	3	
23.	Woman's employment status	ባለፉት 12 ወራት ውስጥ ከቤት ስራ በተጨማሪ ሌላ ስራ ሰርተሻል/ዋል?<br>አስፈላጊ ከሆነ አስረዳ/ጅ :<br><br>አንዳንድ እናቶች ለምሳሌ ንግድ፤የቤተሰቡ እርሻ ላይ መስራት፤የጉሮ አትክልት መሸጥ፤የቀን ስራ መስራት እና የመሳሰሉትን አይነት ስራ ከቤት ስራ በተጨማሪ ይሰራሉ፤እረስዎስ እንደዚህ አይነት ስራ ሰርተዋል?	አዎ	1	
			አልሰራሁም	2	25
24.	Woman's occupation	ስራዎት ምንድን ነው፤<br><br>ለዚህ ጥያቄ መልስ የቤት እመቤት ከሆነ የጥያቄ 23 መልስ አልሰራሁም መሆን አለበት	ነጋዴ	1	
			አርሶ አደር/ገበሬ	2	
			የቤት ሰራተኛ	3	
			ፀሀፊ	4	
			ሌላ፤ግለፅ	99	

4 -የባል መሰረታዊ መረጃ					
**ለጥያቄ ቁጥር 14 የሰጡት መልስ 1 ከሆነ ቀጣዩቹን ጥያቄዎች በመዝለል ወደ ክፍል 6 ይቀጥሉ**					
አስረዳ: ከዚህ በመቀጠል ስለ ትዳር አጋርዎ/ባለቤትዎ ነው የምጠይቀዎት።					
**Nr**	**Variable**	**Questions & filters**	**Coding categories**		**Skip**
25.	Partner's educational level	ባለቤትዎ/የትዳር አጋርዎ ትምህርት ተከታትለዋል?	አዎ	1	
			አልተከታተሉም	2	27
26.	Partner's educational level	የባለቤትዎ/የትዳር አጋርዎ የትምህርት ደረጃ?	የመጀመሪያ ደረጃ	1	
			ሁለተኛ ደረጃ	2	
			ቴክኒክና ሙያ	3	
			ከፍተኛ ደረጃ ትምህርት	4	
27.	Partner's employment status	የባለቤትዎ/የትዳር አጋርዎ ስራ ምንድን ነው?	ነጋዴ	1	
			አርሶ አደር/ገበሬ	2	
			ሹፌር	3	
			የመንግስት ሰራተኛ	4	
			ስራ የሌለው	5	
			ሌላ ግለፅ	99	

Survey number [ ] [ ] [ ] [ ] Data collector ID [ ] [ ]

5 - የቤት ውስጥ ስራን እና ውሳኔዎችን በመወሰን ዙሪያ					
**Nr**	**Variable**	**Questions & filters**	**Coding categories**		**Skip**
**28.**	Decision-making earnings	ማን ነው በበላይነት የቤተሰቡ ገቢ ላይ የሚወስነው?	እኔ	1	
			ባል/የትዳር አጋር	2	
			በጋራ	3	
			ገቢ የለም	4	
			ሌላ ሰው፣ግለፅ	99	
**29.**	Division household chores	ባለቤትዎ የቤት ውስጥ ስራን ያግዞታል ለምሳሌ ልጆችን በመንከባከብ፣ምግብ በማብሰል፣ቤት በማፅዳት እና የመሳሰሉትን..	ያግዘኛል	1	
			አያግዘኝም	2	
**30.**	Presence others	በአሁኑ ሰአት ሌላ ሰው በአካባቢ መኖሩን ያረጋግጡ<br>1. ሌላ ሰው አለ እያዳመጡን ነው<br>2. ሌላ ሰው አለ እያዳመጡን አይደለም<br>3. ሌላ ሰው የለም	ልጆች < 10አመት	1<br>2<br>3	
			ባል	1<br>2<br>3	
		መግቢያ፣በአንድ አንድ የሀገራችን ክፍል ባል ሚስትን መቅጣት/መምታት በባህል እንደ ተገቢ ተደርጎ ይወሰዳል	ሌላ ወንድ	1<br>2<br>3	
			ሌላ ሴት	1<br>2<br>3	
**31.**	Domestic/ sexual violence	በርስዎ አመለካከት ባል ሚስቱን የመቅጣት ወይም የመምታት መብት አለዉ?	አዎ	1	
			የለውም	2	

6 - የቤት ውስጥ ሀብት					
**Nr**	**Variable**	**Questions & filters**	**Coding categories**		**Skip**
**32.**	Household wealth	በቤተሰብዎ/በቤትዎ ውስጥ			
		• በቂ ቀለብ/ምግብ	አለ	1	
			የለም	2	
		• ንፁህ የመጠጥ ውሃ አቅርቦት/ቦንቦ ውሃ	አለ	1	
			የለም	2	
		• መብራት	አለ	1	
			የለም	2	
		• ቴሌቭዥን	አለ	1	
			የለም	2	
		• ሞባይል	አለ	1	
			የለም	2	
		• የእርሻ መሬት	አለ	1	
			የለም	2	
		• ከብቶች(ላም፣በሬ፣በጎች ወይም ፍየል)	አለ	1	
			የለም	2	
**33.**	Relative household wealth	በሀብት ደረጃ እራስዎን ከአካባቢዎ አንፃር ሲያወዳድሩ እንዴት ይገልፁታል	በጣም ሀብታም	1	
			ሀብታም	2	
			ደሃ	3	
			በጣም ደሃ	4	

Survey number [ ] [ ] [ ] [ ] Data collector ID [ ] [ ]

7 - ስነ ተዋልዶ እና የወሊድ መቆጣጠር					
አሁን ደግሞ እስከአሁን ደረስ ሰለነበርዎት እርግዝና እና ስለልጆችዎ እጠይቀዎታለሁ					
**Nr**	**Variable**	**Questions & filters**	**Coding categories**		**Skip**
**34.**	Birth giving	ልጅ ወልደዋል?	አዎ	1	
			አይ	2	36
**35.**	Total number of live births	በሕይወት የተወለዱ ስንት ልጆች አሉዎት			
**36.**	IUFD	ሕይወት የሌለው ልጅ ወልደው ያውቃሉ	አዎ	1	
			አይ	2	38
**37.**	Total number of IUFD	ስንት ጊዜ ነው ይህ የገጠመዎት			
**38.**	Early Neonatal death	በሕይወት ከተወለደ በሃላ በመጀመሪያው ሳምነት የሞተ ልጅስ ነበር?<br><br>ከተወለደ በኃላ ማልቀስ ወይም ሌላ በሕይወት የመኖር ምልክት ካሳየ በኃላ የሞተ ልጅ	አዎ	1	
			አይ	2	40
**39.**	Total number of early neonatal deaths	ስንት ጊዜ ነው ይህስ የገጠመዎት?			
**40.**	Total number of births	በአጠቃላይ ስንት ጊዜ ወልደዋል? ምንም ካልወለዱ *00* በማለት ይመዝግቡ<br>በሕይወት ያልተወለዱትንም ያጠቃልላል(35†37)			
**41.**	Pregnancy	ባሁኑ ሰአት ነፍሰጡ/እርጉዝ ነዎት?	አዎ	1	
			አይደለሁም	2	45
			እርግጠኛ አይደለሁም	98	45
የጥያቄ 40 መልስ "ዜሮ" ሆኖ የጥያቄ 41 መልስ "አይደለሁም" ወይም እርግጠኛ አይደለሁምከሆነ ተጠያቂዋን ደግመው በመጠየቅ ያረጋግጡና ለውጥ ከሌለ መጠይቁን ያቆርጡ፡፡					
**42.**	Completed months of pregnancy	እርግዝናው ስንት ወር ነው?<br>(የጨረሻቸውን ወራት በቻ)	……ወር		
**42a**	ANC Visits	በእርግዝና ወቅት የህክምና ክትትል ስንት ጊዜ አድርገዋል?			
**42b**	Place of ANC	በእርግዝና ወቅት የህክምና ክትትል ያደረጉት የት ነው?	ጤና ኬላ	1	
			ጤና ጣቢያ	2	
			ሆስፒታል	3	
			ሌላ ግለጽ	99	
**43.**	Knowledge EDD	የምትወልጅበትን ቀን ታውቂዋለሽ?	አዎ	1	
			ኤይ አላውቀውም	2	
**44.**	(Un)wanted/ planned/supported pregnancy	እርግዝናው የታቀደበት፤የተፈለገ እና መንከባከብ የምትችሉት ነው?<br><br>**ሁሉንም ምላሾች መዝግቢ**	የታቀደ	1	
			የሚፈለግ	2	
			መንከባከብ የሚቻል	3	
			ከዚያ ውጭ	4	
**45.**	Contraceptive use	የወሊድ መቆጣጠሪያ ወይም የቤተሰብ ምጣኔ ዘዴዎችን ተጠቅመው ያውቃሉ?	አሁን እየተጠቀሙ ነው	1	
			ከዚህ በፊት ተጠቅመዋል	2	
			ተጠቅመው አያውቁም	3	
**46.**	Decision-making MCH	የእርሶዎን እና የልጅዎን ጤንነት በተመለከተ አብዛኛውን ጊዜ ማን ነው የሚወስነው?<br><br>ለምሳሌ እርስዎ ወይም ልጅዎ ቢታመሙ የህክምና እርዳታ ለማግኘትም ሆነ ላለማግኘት ማን ነው እሚወስነው	እኔ	1	
			ባል/የትዳር አጋር	2	
			በጋራ	3	
			ሌላ ሰው፤ግለፅ	99	

Nr	Variables	Filters	1ኛ ልጅ	2ኛ ልጅ	3ኛ ልጅ	4ኛ ልጅ	5ኛ ልጅ	6ኛ ልጅ	Skip
**7 –ልጆችን የተመለከተ ጥያቄ የቀጠለ…….**									
ሁሉንም እርግዝና ይመዝግቡ (በሕይወት ያልተወለዱትንም ያጠቃልላል)፤ለመንታ ልጆች እያንዳንዳቸውን ለየብቻ ይመዝግቡ፤ከ 6 በላይ ልጆች ካሉ ተጨማሪ .									
**47.**	የልጁ ስም								
**48.**	የትውልድ ዘመን								
**49.**	በሕይወት አለ	አለ	1	1	1	1	1	1	**51**
		የለም	2	2	2	2	2	2	
**50.**	በሕይወት ከሌለ ሲሞት እድሜው ስንት ነበር?	እድሜ በደቂቃ/ሰአት/ቀን/ሳምንት/ወራት/አመት							
**51.**	በሕይወት ካለ አሁን እድሜው ስንት ነው?	በጨረሳቸው አመታት ልክ ይመዝግቡ							
**52.**	በእርግዝና ወቅት የህክምና ክትትል ስንት ጊዜ አድርገዋል?								
**53.**	የት ነው የወለዱት?	ቤት	1	1	1	1	1	1	
		የጤና ተቋም	2	2	2	2	2	2	
**ሁሉም ልጆች የተወለዱት ተመሳሳይ ቦታ ከሆነ የሚከተሉትን ጥያቄዎች በቅርብ/መጨረሻ ለወለደችው ልጅ ይጠይቁ**<br>**የምትወልድበት ቦታ ከተቀየረ(ለምሳሌ ቤት ከመውለድ ወደ ጤና ተቋም/ከጤና ተቋም ወደ ቤት መውለድ) ቀጣዮቹን ጥያቄዎች ለነዚህ ለውጥ ለተደረገባቸው ልጆች ይጠይቁ::**<br>**በጤና ተቋም ለወለዱ 54፤**<br>**ቤት ለወለዱ 55**									54<br>55
**54.**	ጤና ተቋም ለምን ወለድሽ?<br>ሁሉንም ምክንያቶች የመዝግቡ	ምቹ ስለሆነ	1	1	1	1	1	1	57
		የግል ነፃነት ስለሚሰማኝ	2	2	2	2	2	2	
		ከዚህ ቀደም የነበረው እርግዝና ላይ ችግር ስለነበ	3	3	3	3	3	3	
		በምጥ ወቅት ችግር ስለነበር	4	4	4	4	4	4	
		ባል ወይም ቤተሰብ ስለወሰኑ	5	5	5	5	5	5	
		ወጭ ስለሌለው	6	6	6	6	6	6	
		ሌላ ግለፅ	99	99	99	99	99	99	

Survey number [ ] [ ] [ ] [ ] Data collector ID [ ] [ ]

	Variables	Filters	1ኛ ልጅ	2ኛ ልጅ	3ኛ ልጅ	4ኛ ልጅ	5ኛ ልጅ	6ኛ ልጅ	Skip
**55.**	ቤት ከሆነ የወለዱት ለምን ቤት ወለዱ? ሁሉንም ምክንያቶች የመዝግቡ	ምቹ ስለሆነ	1	1	1	1	1	1	
		የግል ነፃነት ስለሚሰማኝ	2	2	2	2	2	2	
		ቤት ውስጥ ባህላዊ ነገሮች ስለሚደረጉልኝ....	3	3	3	3	3	3	
		ባል ወይም ቤተሰብ ስለወሰኑ	4	4	4	4	4	4	
		ችግር ስላልነበር	5	5	5	5	5	5	
		ወጭ ስለሌለው	6	6	6	6	6	6	
		ሌላ ግለፅ	99	99	99	99	99	99	
**56.**	ቤት ከሆነ የወለዱት ለምን ነበር በጤና ተቋም ያልወለዱበ? ሁሉንም ምክንያቶች የመዝግቡ	ክፍያው ብዙ ስለሆነ	A	A	A	A	A	A	
		ብዙ ጊዜ የጤና ተቋሙ ዝግ ስለሚሆን/ወደ ሌላ ጤና ተቁም ሪፈር የመባል እድል ስላ	B	B	B	B	B	B	
		ጤና ተቋሙ ሩቅ ስለሆነ እና ትራንስፖርት ስለሌለ	C	C	C	C	C	C	
		በጤና ተቋሙ ስለማልተማመን	D1	D1	D1	D1	D1	D1	
		አገልግሎቱ ጥሩ ስላልሆነ	D2	D2	D2	D2	D2	D2	
		አገልግሎቱን የሚሰጡ ሴቶች ስላልሆኑ	E	E	E	E	E	E	
		ባል ወይም ቤተሰብ ፈቃደኛ ስላልሆኑ	F	F	F	F	F	F	
		የቤተሰቡ አባላት ፈቃደኛ ስላልሆኑ	G	G	G	G	G	G	
		የግል ነፃነት ስለሌለው	H	H	H	H	H	H	
		No cultural rituals	I	I	I	I	I	I	
		ችግር ስላልነበር	J	J	J	J	J	J	
		ኦፕሬሽን በመፍራት	K	K	K	K	K	K	
		ሌላ ግለፅ	99	99	99	99	99	99	
**57.**	በምን መንገድ ነው የወለድሽው	በማሕፀን	1	1	1	1	1	1	
		VE	2	2	2	2	2	2	
		በኦፕሬሽን	3	3	3	3	3	3	
**58.**	ችግር ነበር	አዎ ነበር	1	1	1	1	1	1	
		አልነበረም	2	2	2	2	2	2	**63**

Survey number [ ] [ ] [ ] [ ] Data collector ID [ ] [ ]

	Variables	Filters	1ኛ ልጅ	2ኛ ልጅ	3ኛ ልጅ	4ኛ ልጅ	5ኛ ልጅ	6ኛ ልጅ	Skip
**59.**	ከነበር ምንድን ነው የተከሰተው<br>ሁሉንም ምክንያቶች የመዝግቡ	ደም መፍሰስ	1	1	1	1	1	1	
		ምጥ መራዘም	2	2	2	2	2	2	
		የማህፀን መጥበብ	3	3	3	3	3	3	
		ደም ግፊት	4	4	4	4	4	4	
		Puerperal infection	5	5	5	5	5	5	
		በማሕፀን ውስጥ የፅንሱ መታፈን	6	6	6	6	6	6	
		ፅንሱ በማሕፀን ውስጥ መሞት	7	7	7	7	7	7	
		ሌላ ግለፅ	99	99	99	99	99	99	
								ጤና ተቋም ከሆነ የወለዱት	**63**
**60.**	ችግር ከነበር የህክምና እርዳታ ለማግኘት ሞክረዋል	አዎ	1	1	1	1	1	1	**61**
		አይ አልሞከርኩም	2	2	2	2	2	2	**62**
**61.**	የህክምና እርዳታ ለማግኘት ከሞከሩ የት ነው	የልምድ አዋላጅ	1	1	1	1	1	1	**63**
		የጤና ተቋም	2	2	2	2	2	2	
		ሌላ ግለፅ	99	99	99	99	99	99	
**62.**	የሕክምና እርዳታ ለማግኘት ካልሞከሩ ለምን<br>ሁሉንም ምክንያቶች የመዝግቡ	ክፍያው ብዙ ስለሆነ	A	A	A	A	A	A	
		ብዙ ጊዜ የጤና ተቋሙ ዝግ ስለሚሆን…	B	B	B	B	B	B	
		ጤና ተቋሙ ሩቅ ስለሆነ እና ትራንስፖርት ስለሌለ	C	C	C	C	C	C	
		በጤና ተቋሙ ስለማልተማመን	D1	D1	D1	D1	D1	D1	
		አገልግሎቱ ጥሩ ስላልሆነ	D2	D2	D2	D2	D2	D2	
		አገልግሎቱን የሚሰጡ ሴቶች ስላልሆኑ	E	E	E	E	E	E	
		ባል ወይም ቤተሰብ ፈቃደኛ ስላልሆኑ	F	F	F	F	F	F	
		አስፈላጊ ስላልሆነ	G	G	G	G	G	G	
		ሰላልተለመደ	H	H	H	H	H	H	
		ሌላ ግለፅ	99	99	99	99	99	99	

8 - ከእርግዝና ጋር በተያያዘ ሊከሰቱ ስለሚችሉ ችግሮች/አደገኛ ምልክቶች ያለ እውቀት

በእርግዝና ወቅት የህክምና ክትትል አድርገው የማያውቁ ከሆነ ወደ ጥያቄ ቁጥር 64 ይቀጥሉ

Nr	Variable	Questions & filters	Coding categories		Skip
63.	Health education complications	በእርግዝና ወቅት የህክምና ክትትል በሚያደርጉበት ጌዜ ከእርግዝና ጋር ተያያዥ ስለሆኑ ችግሮች/ሊከሰቱ ስለሚችሉ አደገኛ ምልክቶች ተነግሮወታል?<br>ግልፅ ካልሆነ አስረዳ፤በእርግዝና ወቅት ከእርግዝና ጋር በተያያዘ በአንቺ ወይም በልጅሽ ላይ ሊከሰቱ የሚችሉ የጤና ችግሮች ናቸው።	ተነግሮኛል	1	
			አልተነገረኝም	2	
64.	Knowledge pregnancy-complications	በእርግዝና ወቅት ሊከሰቱ ከሚችሉ ችግሮች/አደገኛ ምልክቶች ውስጥ የሚያውቁቱን ሊነግሩኝ ይችላሉ?	ደም መፍሰስ	A	
			ከማሕፀን ውሃ መፍሰስ	B	
			ከፍተኛ ራስ ምታት	C	
			አይን ሸዥ ማለት	D	
			ትኩሳት	E	
			ሆድ ህመም/ጊዜውን ያልጠበቀ ምጥ	F	
			የፅንሱ እንቅስቃሴ መቀነስ	G	
			ሰውነት ማበጥ	H	
			ሌላ ግለፅ	99	

ተጠያቂዋ በእርግዝና ወቅት ስለሚከሰቱ ችግሮች የማያውቁ ከሆነ ወደ ቀጣዩ ጥያቄ ከመሄድዎ በፊት ይግለፁላቸው

በእርግዝና ወቅት የህክምና ክትትል አድርገው የማያውቁ ከሆነ ወደ ጥያቄ ቁጥር 66 ይቀጥሉ

Nr	Variable	Questions & filters	Coding categories		Skip
65.	Health education birth preparedness	በእርግዝና ወቅት የህክምና ክትትል በሚያደርጉበት ጊዜ ከወሊድ በፊት ማድረግ ስላለብዎት ቅድመ ዝግጅት ተገልፆሎታል<br>ግልፅ ካልሆነ አስረዳ:ከወሊድ በፊት ሊደረግ የሚገባ ቅድመ ዝግጅት ስባል የሚከተሉትን ያካትታል።<br>1. በእርግዝና ወቅት በቅድሚያ የመውለጃ ቦታን መወሰን፡-ቤት ከሆነ የሰለጠነ አዋላጅ/ጤና ተቋም ከሆነ ችግር እንኩዋን ቢፈጠር በኦፐሬሽን ማዋለድ የሚችል ተቋም ለይቶ ማዘጋጀት<br>2. በምን አይነት መጓጓዣ ወደ ጤና ተቋሙ መሄድ እንዳለብዎ፤ለትራንስፖርትና ለያጋጥሙ ለሚችሉ የህክምና ወጭዎች ሊከፈል የሚችል ብር መቆጠብ<br>3. ብዙ ደም መፍሰስ ቢያጋጥምዎ ያንን ለመተካት ደም የሚለግስ ሰው ቀድሞ ማዘጋጀት	ተገልፆልኛል	1	
			አልተገልፀልኝም	2	
66.	BP Advance planning place of delivery	በእርግዝናዎ ወቅት ቀድመው የት መውለድ እንዳለብዎት/የመውለጃ ቦታን ወስነሽ ነበር?	አዎ	1	
			አልወሰንኩም	2	73
67.	BP planned place of delivery	የት ለመውለድ ነበር ቀድመው ያቀዱት	ቤት	1	68
			በጤና ተቋም	2	69
			ሌላ ግለፅ	99	
68.	BP trained BA	ቤት ከሆነ በቅድሚያ የሰለጠነ አዋላጅ አዘጋጅተው ነበር	አዘጋጅቸ ነበር	1	
			አላዘጋጀሁም	2	
69.	BP HF	ችግር እንኩዋን ቢፈጠር በኦፐሬሽን ማዋለድ የሚችል ተቋም ለይቶ አዘጋጅተው	አዘጋጅቸ ነበር	1	
			አላዘጋጀሁም	2	
70.	BP transport	በምን አይነት መጓጓዣ ወደ ጤና ተቋሙ መድረስ እንዳለብዎ ቀድመው አቅደዋል	አቅጃለሁ	1	
			አላቀድኩም	2	
71.	BP money	ለያጋጥሙ ለሚችሉ የህክምና ወጭዎች ሊከፈል የሚችል ብር መቆጠብ	ቆጥቤያለሁ	1	
			አልቆጠብኩም	2	
72.	BP blood donor	ደም መለገስ የሚችል ሰው ቀድመው አዘጋጅተው ነበር	አዘጋጅቸ ነበር	1	
			አላዘጋጀሁም	2	

Survey number [ ] [ ] [ ] [ ] Data collector ID [ ] [ ]

9 - ለጉዳት ተጋላጭነት ያለ ግንዛቤ					
**Nr**	**Variable**	**Questions & filters**	**Coding categories**		**Skip**
**73.**	Perceived risk home delivery -mother	የት መውለድዎ በበለጠ የእርስዎን ጤና ለጉዳት ያጋልጠኛል ብለው ያስባሉ?	ቤት	1	
			ጤና ተቋም	2	
**74.**	Perceived risk home delivery - baby	የት መውለድዎ በበለጠ የልጅዎን ጤና ለጉዳት ያጋልጠዋል ብለው ያስባሉ?	ቤት	1	
			ጤና ተቋም	2	

10 - የጤና አገልግሎት እና የአካባቢ ሁኔታ					
አስረዳ/ጅ: አሁን ደግሞ በአካባቢዎ ስለሚገኙ የጤና ተቋማት እና እርስዎ ያልዎትን የግልጋሎት ልምድ እጠይቅዎታለሁ					
**Nr**	**Variable**	**Questions & filters**	**Coding categories**		**Skip**
**75.**	Travel time Health Post	ከቤትዎት እስከ ጤና ኬላው ድረስ ለመንገዝ ምን ያክል ጊዜ ይፈጃል?	ደቂቃ		
			ሰዓት		
		አጠቃለይ ለመድረስ የሚፈጀውን ጊዜ በደቂቃ እና/ወይም በሰዓት ይመዝግቡ	ጤና ኬላ የለም	98	
**76.**	Travel time Health Center	ከቤትዎት እስከ ጤና ጣቢያ ድረስ ለመንገዝ ምን ያክል ጊዜ ይፈጃል?	ደቂቃ		
			ሰዓት		
			ጤና ጣቢያ የለም	98	
**77.**	Travel time Hospital	ከቤትዎት እስከ ባቅራቢያዎ ወደሚገኝ ሆስፒታል ለመንገዝ ምን ያክል ጊዜ ይፈጃል?	ደቂቃ		
			ሰዓት		
**78.**	Emergency transport	ምጥ ቤት ከጀመረ በሆላ ጤና ተቋም ለመድረስ ትራንስፖርት ማግኘት ምን ያክ ከባድ ወይም ቀላል ነው	በጣም ቀላል	1	
			ቀላል	2	
			ከባድ	3	
			በጣም ከባድ	4	
**79.**	Emergency mode of transport	ቤት በሚወልዱበት ወቅት ችግር ቢፈጠር ጤና ተቋም ለመድረስ አብዛኛውን መንገድ በምን ይጓዛሉ	በእግር ሰው ተሸክሞኝ	1	
			በፈረስ	2	
			በህዝብ ትራንስፖርት	3	
			በግል ትራንስፖርት	4	

11 - ለእናቶችና ለሕፃናት የሚሰጥ የጤና አገልግሎት ጥራት					
**Nr**	**Variable**	**Questions & filters**	**Coding categories**		**Skip**
**80.**	Nearest hospital	ለመኖሪያ ቤትዎ የሚቀርበው ሆስፒታል የቱ ነው?	አጣጥ ሆስፒታል	A	
			ቡታጅራ ሆስፒታል	B	
			መርሲ ሆስፒታል	M	
			ሌላ ግለፅ	99	
**81.**	Utilization hospital	በዚህ ሆስፒታል ተጠቅመው ያውቃሉ?	አዎ	1	
			አላውቅም	2	84
**82.**	Utilization MCH at hospital	የእናቶችና የሕፃናት ጤና አገልግሎት በዚህ ሆስፒታል ተጠቅመው ያውቃሉ ?	አዎ	1	
			አላውቅም	2	84
**83.**	Utilization types of MCH services at hospital	የትኛውን የእናቶችና የሕፃናት ጤና አገልግሎት ነው የተጠቀሙት?<br>የቅድመወሊድ ክትትል፤በርግዝና ወቅት የሚደረግ የህክምና አገልግሎት<br>አልትራሳውንድ፤የፅንሱን አቀማመጥ፤እድገት በቴሌቭዥን ማየት<br>የማዋለድ አገልግሎት፤በሰለጠኑ ባለሙያወች ማዋለድ<br>ድህረ ወሊድ አገልግሎት፤ከወለድሽ በሆላ ለልጅ እና ለእናት የሚሰጥ የህክምና አገልግሎት<br>ሁሉን ይመዝግቡ	የቅድመ ወሊድ ክትትል	A	85
			አልትራሳውንድ	B	
			የማዋለድ አገልግሎት	C	
			ድህረ ወሊድ አገልግሎት	D	

Survey number [ ] [ ] [ ] [ ] Data collector ID [ ] [ ]

Nr	Variable	Questions & filters	Coding categories		Skip
**84.**	Reasons for non-utilization (MCH at) hospital	ከላይ የተጠቀሱትን አገልግሎቶች በሆስፒታሉ ተጠቅመው የማያውቁ ከሆነ ምክንያትዎ ምንድን ነው?<br><br>ሁሉን ምክንያቶች ይመዝግቡ	ክፍያው ብዙ ስለሆነ	A	97
			ብዙ ጊዜ የጤና ተቋሙ ዝግ ስለሚሆን…	B	
			ጤና ተቋሙ ሩቅ ስለሆነ እና ትራንስፖርት ስለሌለ	C	
			በጤና ተቋሙ ስለማልተማመን	D1	
			አገልግሎቱ ጥሩ ስላልሆነ	D2	
			አገልግሎቱን የሚሰጡ ሴቶች ስላልሆኑ	E	
			ባል ወይም ቤተሰብ ፈቃደኛ ስላልሆኑ	F	
			አስፈላጊ ስላልሆነ	G	
			ስላልተለመደ	H	
			ሌላ ግለፅ	99	
ከላይ የተጠቀሱትን አገልግሎቶች በሆስፒታሉ ተጠቅመው የሚያውቁ ከሆነ ከሚከተሉት ነገሮች አንፃር ጥራቱን እንዴት ያዩታል					
**85.**	Perceived quality of care MCH hospital - overall	• አጠቃላይ አገልግሎት	ጥሩ	4	
			አጥጋቢ	3	
			በቂ	2	
			ዝቅተኛ	1	
**86.**	Perceived quality of care MCH hospital - supplies	• የመድሐኒትና የሕክምና ቁሳቁስ አቅራቦት	ጥሩ	4	
			አጥጋቢ	3	
			በቂ	2	
			ዝቅተኛ	1	
**87.**	Perceived quality of care MCH hospital – waiting times	• አገልግሎት ለማግኘት የሚጠብቁት ሰዓት	ጥሩ	4	
			አጥጋቢ	3	
			በቂ	2	
			ዝቅተኛ	1	
**88.**	Perceived quality of care MCH hospital – quality staff	• የሰራተኞች የሙያ ብቃት	ጥሩ	4	
			አጥጋቢ	3	
			በቂ	2	
			ዝቅተኛ	1	
**89.**	Perceived quality of care MCH hospital – friendliness staff	• የሰራተኞች ቀረቤታ	ጥሩ	4	
			አጥጋቢ	3	
			በቂ	2	
			ዝቅተኛ	1	
**90.**	Perceived quality of care MCH hospital – respect preferences	• ለተገለገይ ያላቸው አክብሮት	ጥሩ	4	
			አጥጋቢ	3	
			በቂ	2	
			ዝቅተኛ	1	
**91.**	Perceived quality of care MCH hospital – privacy	• የግል ነፃነት	ጥሩ	4	
			አጥጋቢ	3	
			በቂ	2	
			ዝቅተኛ	1	
**92.**	Perceived quality of care MCH hospital - hygiene	• ንፅህና	ጥሩ	4	
			አጥጋቢ	3	
			በቂ	2	
			ዝቅተኛ	1	

Nr	Variable	Questions & filters	Coding categories		Skip
93.	Perceived quality of care MCH hospital - language	• በራስ ቋንቋ መጠቀም ይቻላል	ይቻላል	1	
			አይቻልም	2	
94.	Perceived quality of care MCH hospital - discrimination	• አንዳንድ ተገልጋዮች ይግለላሉ ወይ<br><br>**አስረዳ/ጅ : አንድ አንድ ጊዜ አንድ አንድ ሰዎች በዘር፤በኑሮቸው ደረጃ ወይም ባላቸው ህመም ምክንያት(HIV) በሌሎች ሰዎች ዘንድ መገለል ሊደርስባቸው ይችላል.**	አዎ	1	
			አይገለሉም	2	
95.	Perceived quality of care MCH hospital - cost	• ክፍያ	ተመጣጣኝ	1	
			የልተመጣጠነ	2	
96.	Recommendation MCH Hospital	ለሌሎች ነፍሰጡር እናቶች አገልግሎቱን እንዲያገኑ ይመክራሉ	እመከራለሁ	1	
			አልመክርም	2	

12 A - የነፈሰጡር እናቶች ማረፊያ ቤት-ተጠቃሚ ላልሆኑት					
**Nr**	**Variable**	**Questions & filters**	**Coding categories**		**Skip**
97.	Knowledge MWH	ስለ ነፈሰጡር እናቶች ማረፊያ ቤት ሰምተው ያውቃሉ	አዎ	1	
			አላውቅም	2	101
ከዚህ በመቀጠል የነፈሰጡር እናቶችን ማረፊያ ቤትን በተመለከተ በሰፊው ለሁሉም እናቶች ገለጻ ያድርጉ፤የነፈሰጡር እናቶች ማረፊያ ቤት ማለት በወሊድ ወቅት ችግር ሊገጥማቸው ይችላል ተብሎ የሚገመቱ እናቶች የመውለጃ ቀናቸው ሁለት ውይም ሶስት ሳምንት ሲቀረው ችግር ቢከሰት በኦፕሬሽን ማዋለድ በሚችል የጤና ተቋም ውስጥ በተሰራ ማረፊያ ቤት ተቀምጠው እስኪወልዱ ድረስ የሚያርፉበት ቤት ነው ምጥ ሲጀምር በጤና ተቋሙ እንዲወልዱ ይደረጋል ማለት ነው					
98.	Knowledge MWH: availability	በእርስዎ አካባቢ የነፈሰጡር እናቶች ማረፊያ ቤት አገልግሎት የሚሰጥ ተቋም አለ	አለ	1	
			የለም	2	101
99.	Location MWH	የነፈሰጡር እናቶች ማረፊያ ቤቱ የት ነው ያለው	አጣጥ ሆስፒታል	A	
			መርሲ ሆስፒታል	M	
			ሌላ ግለፅ	99	
100.	Stay MWH	በሆስፒታል ውስጥ በሚገኝ የነፈሰጡር እናቶች ማረፊያ ቤት ተገልግለዉ ያውቃሉ	አውቃለሁ	1	**116**
			አላውቅም	2	

101.	Knowledge MWH: advantages	በነፈሰጡር እናቶች ማረፊያ ቤት መቆየቱ ጥቅሙ ምንድን ነው ብለው ያስባሉ<br><br>ሁሉንም ምክንያቶች ይመዝግቡ	በቅርበት ህክምና ለማገኘት	1	
			የእናት ህይወት ያድናል	2	
			የልጅ ህይወት ያድናል	3	
			ከወሊድ በፊት እረፍት ለማድረግ	4	
			ሌላ ግለፅ	99	
			ምክንያቱን አለማወቅ	98	
**ተጠያቂዋ ጥቅሙ ካልገባቸው እባክዎን ያስረዱ**					
102.	Likeliness stay MWH	ለአሁኑ ወይም ለቀጣዩ እርግዝናዎ በነፈሰጡር እናቶች ማረፊያ ቤት ከመውለጃዎ ጊዜ ከ2 - 4 ሳምንት ቀደም ብለሽ ልትቆይ የምትችይበት እድል ምን ያክል ነው(መቆየት አለብሽ ብትባይ)	በጣም ሰፊ እድል	1	
			ሰፊ እድል	2	
			ጠባብ እድል	3	
			በጣም ጠባብ እድል	4	
**አስረዳ፡በነፈሰጡር እናቶች ማረፊያ ቤት ከመውለጃዎ ጊዜሽ ከ2 - 4 ሳምንት ቀደም ብለሽ መቆየት አለብሽ ብትባይ ወደ ማረፊያ ቤቱ ከመምጣት/አገልግሎቱን ከማግኘት ምን ሊያግድሽ ይችላል**					
103.	Barrier MWH transport	• ወደ ነፈሰጡር እናቶች ማረፊያ ቤት በትራንስፖርት ለመምጣት	አቅሜ ይፈቅድልኛል	1	
			አቅሜ አይፈቅድልኝም	2	

Nr	Variable	Questions & filters	Coding categories		Skip
**104.**	Barrier MWH food	• በምትቆይበት ጊዜ ለምግብ/ለቀለብ ያለው የገንዘብ ወጭ	አቅሜ ይፈቅድልኛል	1	
			አቅሜ አይፈቅድልኝም	2	
**105.**	Barrier MWH utensils	• በምትቆይበት ጊዜ ለምግብ ማብሰያ የሚሆኑ የራስሽን እቃዎች ይዞ ማምጣት	የሚቻል	1	
			የማይቻል	2	
**106.**	Barrier MWH waiting time respondent	• ከመውለደሽ በፊት ከ 2 እስከ 4ሳምንታት ለሚያክል ጊዜ ከቤት ርቀሽ በማረፊያ ቤቱ መቆየት	የሚቻል	1	
			የማይቻል	2	
**107.**	Barrier MWH waiting time attendant	• ለሁለት ሳምንትና ከዚያ በላይ ለሚያክል ጊዜ አብሮ በማረፊያ ቤቱ በመሆን ሊያስታምምሽ የሚችል ሰው ማግኘት	የሚቻል	1	
			የማይቻል	2	
**108.**	Barrier MWH children at home	• አንች በማረፊ ቤቱ በምትቆይበት ጊዜ ቤትሽ ልጆችሽን የሚንከባከብ ቤተሰብ ወይም ዘመድ ማግኘት	የሚቻል	1	
			የማይቻል	2	
**109.**	Barrier MWH household chores	• አንች በማረፊ ቤቱ በምትቆይበት ጊዜ ቤትሽ ውስጥ ያለውን ስራ የሚያግዝ ዘመድ ወይም ቤተሰብ ማግኘት	የሚቻል	1	
			የማይቻል	2	
**110.**	Barrier MWH loss from productivity activity	• ለረጅም ጊዜ ከስራሽ መራቅ(ከቤት ውስጥ ስራ ውጭ)	የሚቻል	1	
			የማይቻል	2	
**111.**	Barrier MWH attendant loss productivity	• አስታማሚሽ ከስራው/ዋ ለረጅም ጊዜ መራቅ	የሚቻል	1	
			የማይቻል	2	
**112.**	Barrier MWH dependent child	• ልጆችሽን ከአንች ጋር ወደ ነፈሰጡር ማረፊያ ቤቱ ይዞ መምጣት	አስፈላጊ	1	
			አላስፈላጊ	2	
**113.**	Barrier MWH family visits	• ጠያቂ ቤተሰብ መነሩ	አስፈላጊ	1	
			አላስፈላጊ	2	
**114.**	Other barriers MWH	በነፈሰጡር እናቶች ማቆያ ቤት ከመቆየት ሊያግድ የሚችል ሌላ እሚያስቡት ምክንያት አለ	አለ	1	
			የለም	2	መጨረሻ
**115.**	Other barriers specified	አለ ካሉ እባክዎትን ያስረዱ<br>በነፈሰጡር እናቶች ማቆያ ቤት ከመቆየት ሊያግድ የሚችል ሌላ እሚያስቡት ምክንያት አለ		99	መጨረሻ

12 B-የነፈሰጡር እናቶች ማቆያ ቤት-ተጠቃሚ ለሆኑ					
**Nr**	**Variable**	**Questions & filters**	**Coding categories**		**Skip**
**116.**	Location stay MWH	የትኛው የነፈሰጡር እናቶች ማቆያ ቤት ነው የቆዩት ?	አጣጥ ሆስፒታል	A	
			ሌላ ግለፅ	M	
			መርሲ ሆስፒታል	99	
**117.**	Reason admission MWH	የነፈሰጡር እናቶች ማቆያ ቤት እንዲቆዩ ያደረገዎት ምክንያት ምንድን ነው?<br><br>ተጠያቂዋ ምክንያቱን እማያውቁት ከሆነ ሱፐርቫይዘሩ ምክንያቱን ከካርዱ ላይ ይመልከት	ከዚህ ቀደም በቀዶ ጥገና መውለድ	1	
			ከዚህ ቀደም ሽንት ወይም ሰገራን የመቆጣጠር ችግር ኖሮባት በህክምና የተስተካከለላት	2	
			መንታ ማርገዝ	3	
			ከ 6 ጊዜ በላይ መውለድ	4	
			ከዚህ ቀደም በማሕፀን ውስጥ የፅንሱ መሞት የጋጠማጥ	5	
			የፅንሱ አቀማመጥ ጥሩ ካልሆነ	6	
			ፅንሱ በግሩ ሲመጣ	7	
			ከወሊድ በፊት ደም መፍሰስ ስኖር	8	
			ደም ማነስ	9	
			Polyhydramnios	10	
			ደም ግፊትና ሌላ የጤና ችግር	11	
			መኖሪያቸው ከሆስፒታሉ በጣም መራቅ	12	
			ሌላ ግለፅ	99	
**118.**	Referral to MWH	ወደ ነፈሰጡር እናቶች ማቆያ ቤት እንዲሄዱ የተነገርዎት ከየት ነው	በጤና ኤክስቴንሽን ባለሙያ	1	
			ከጤና ኬላ	2	
			ከጤና ጣቢያ	3	
			ከሆስፒታል	4	
			ሌላ ግለፅ	99	
**119.**	Duration stay MWH	ለምን ያክል ጊዜ በነፈሰጡር እናቶች ማቆያ ቤት ቆዩ?<br><br>የቆዩበትን ጊዜ በሙሉ ወይም በክፍልፋይ ሳምንታት ይግለጹ			
**120.**	Decision-making MWH	በነፈሰጡር እናቶች ማቆያ ቤት እዲቆዩ ማን ወሰነ	እኔ	1	
			ባለቤቴ	2	
			በጋራ	3	
			ሌላ ግለፅ	99	
**121.**	Attendant MWH	በነፈሰጡር እናቶች ማቆያ ቤት በሚቆዩበት ወቅት አስታማሚዎ ማን ነበር	ባለቤትዎ	1	
			ዘመድ/ሌላ የቤተሰቡ አባል	2	
			ሌላ ግለፅ	99	
**122.**	Financial support MWH	በነፈሰጡር እናቶች ማቆያ ቤት በሚቆዩበት ወቅት የገንዘብ ድጋፍ ከማን ነበር የሚያገኙት	ከራሴ	1	
			ባለቤቴ	2	
			ዘመድ/ሌላ የቤተሰቡ አባል	3	
			ሌላ ግለፅ	99	

Nr	Variable	Questions & filters	Coding categories		Skip
123.	Social support MWH	በነፈሰጡር እናቶች ማቆያ ቤት በሚቆዩበት ወቅት? (ልጆችን በመንከባከብ፤የቤት ውስጥ ስራዎችን በመስራት እና በሞራል እገዛ የሚያደርግልዎ ማን ነው)	ባለቤቴ	1	
			ዘመድ/ሌላ የቤተሰቡ አባል	2	
			ሌላ ግለፅ	99	
124.	Advantages MWH	በነፈሰጡር እናቶች ማቆያ ቤት መቆየቱ ጥቅሙ ምንድን ነው ብለው ያስባሉ	በቅርበት ህክምና ለማገኘት	1	
			የእናት ህይወት ያድናል	2	
			የልጅ ህይወት ያድናል	3	
			ከወሊድ በፊት እረፍት ለማድረግ	4	
			ሌላ ግለፅ	99	
አስረዳ፦እባክዎት በቀጣዮቹ ነገሮች ላይ ያልዎትን አስተያየት ይግለፁልን አገልግሎቱን ለማሻሻል ስለሚረዳን ሳትሳቀቂ ንገሪኝ					
125.	Barrier MWH transport	• ወደ ነፈሰጡር እናቶች ማቆያ ቤት ለመምጣት ያለው ትራንስፖርት	አቅሜ ይፈቅድልኛል	1	
			አቅሜ አይፈቅድልኝም	2	
126.	Barrier MWH food	• በሚቆዩበት ጊዜ ለምግብ ያለው ወጭ	አቅሜ ይፈቅድልኛል	1	
			አቅሜ አይፈቅድልኝም	2	
127.	Barrier MWH utensils	• በሚቆዩበት ጊዜ ለምግብ ማብሰያ የሚሆኑ የራስ እቃ መምጣት	የሚቻል	1	
			የማይቻል	2	
128.	Barrier MWH waiting time respondent	• ከወሊድ በፊት ከ2-4ሳምንታት ያክል መቆየት	የሚቻል	1	
			የማይቻል	2	
129.	Barrier MWH waiting time attendant	• ለሁለት ሳምንት ያክል አብሮ የሚሆን አስታማሚ ማግኘት	የሚቻል	1	
			የማይቻል	2	
130.	Barrier MWH children at home	• ልጆችን የሚንከባከብ ቤተሰብ/ዘመድ ማግኘት	የሚቻል	1	
			የማይቻል	2	
131.	Barrier MWH household chores	• ቤት ውስጥ ያለውን ስራ የሚያግዝ ዘመድ/ቤተሰብ ማግኘት	የሚቻል	1	
			የማይቻል	2	
132.	Barrier MWH loss from productivity activity	• ፈያሞ ከቤት መራቅ የማይታሰብ ነው	የሚቻል	1	
			የማይቻል	2	
133.	Barrier MWH attendant loss productivity	• አስታማሚዎ ከስራው/ዋ ለረጅም ጊዜ መራቅ አትችልም/አይችልም	የሚቻል	1	
			የማይቻል	2	
134.	Barrier MWH dependent child	• ልጆችን አንድ ላይ ወደ ነፈሰጡር ማቆያ ቤቱ ይዞ መምጣት	አስፈላጊ	1	
			አላስፈላጊ	2	
135.	Barrier MWH family visits	• ጠያቂ ቤተሰብ መነሩ	አስፈላጊ	1	
			አላስፈላጊ	2	
136.	Other barriers MWH	በነፈሰጡር እናቶች ማቆያ ቤት ከመቆየት ሊያግድ የሚችል ሌላ እሚያስበት ምክንያት አለ	አለ	1	
			የለም	2	
137.	Other barriers specified	አለ ካሉ እባክዎትን ያስረዱ		99	
		የነፈሰጡር እናቶች ማቆያ ቤት ከሚከተሉት ነገሮች አንፃር እንዴት ይገመግሙታል አገልግሎቱን ለማሻሻል ስለሚረዳን ሳትሳቀቂ ንገሪኝ			

Nr	Variable	Questions & filters	Coding categories		Skip
**138.**	Perceived quality MWH facilities	• ቤቱ፤ ያሉት የመገልገያ ቁሳቁስ	ጥሩ	1	
			አጥጋቢ	2	
			በቂ	3	
			አነስተኛ	4	
**139.**	Perceived quality MWH hygiene	• ንፅሕናውስ	ጥሩ	1	
			አጥጋቢ	2	
			በቂ	3	
			አነስተኛ	4	
**140.**	Perceived quality MWH facilities	• የግል ነፃነት	ጥሩ	1	
			አጥጋቢ	2	
			በቂ	3	
			አነስተኛ	4	
**141.**	Perceived quality MWH support women	• እርስ በርስ መደጋገፍ	ጥሩ	1	
			አጥጋቢ	2	
			በቂ	3	
			አነስተኛ	4	
**142.**	Perceived quality MWH space attendants	• ለአስታማሚ እና ለጠያቂ ያለው ቦታ	ጥሩ	1	
			አጥጋቢ	2	
			በቂ	3	
			አነስተኛ	4	
**143.**	MWH areas improvemen t	መሻሻል አለበት የሚሉት ነገር አለ			
**144.**	Recommend ation MWH	ሌላ ነፈሰጡር እናቶችየነፈሰጡር እናቶች ማቆያ ቤትን እነዲጠቀሙ ይመክራሉ?	እመክራለሁ	1	መጨረሻ
			አልመክርም	2	
